# Evolution of Phenotypic Variance Provides Insights into the Genetic Basis of Adaptation

**DOI:** 10.1093/gbe/evae077

**Published:** 2024-04-15

**Authors:** Wei-Yun Lai, Viola Nolte, Ana Marija Jakšić, Christian Schlötterer

**Affiliations:** Institut für Populationsgenetik, Vetmeduni Vienna, Vienna, Austria; Vienna Graduate School of Population Genetics, Vetmeduni Vienna, Vienna, Austria; Institut für Populationsgenetik, Vetmeduni Vienna, Vienna, Austria; Institut für Populationsgenetik, Vetmeduni Vienna, Vienna, Austria; Vienna Graduate School of Population Genetics, Vetmeduni Vienna, Vienna, Austria; Present address: École polytechnique fédérale de Lausanne, Lausanne, Switzerland; Institut für Populationsgenetik, Vetmeduni Vienna, Vienna, Austria

**Keywords:** phenotypic variance, temperature adaptation, *Drosophila simulans*, experimental evolution

## Abstract

Most traits are polygenic, and the contributing loci can be identified by genome-wide association studies. The genetic basis of adaptation (adaptive architecture) is, however, difficult to characterize. Here, we propose to study the adaptive architecture of traits by monitoring the evolution of their phenotypic variance during adaptation to a new environment in well-defined laboratory conditions. Extensive computer simulations show that the evolution of phenotypic variance in a replicated experimental evolution setting can distinguish between oligogenic and polygenic adaptive architectures. We compared gene expression variance in male *Drosophila simulans* before and after 100 generations of adaptation to a novel hot environment. The variance change in gene expression was indistinguishable for genes with and without a significant change in mean expression after 100 generations of evolution. We suggest that the majority of adaptive gene expression evolution can be explained by a polygenic architecture. We propose that tracking the evolution of phenotypic variance across generations can provide an approach to characterize the adaptive architecture.

**Significance**

It is widely accepted that most complex traits have a polygenic basis. Nevertheless, it is difficult to predict which of these loci are responding to selection when a population is exposed to a new selection regime. To address this situation, we propose to infer the adaptive architecture for traits by tracking the evolution of their phenotypic variance during adaptation to a new environment. As a case study, we analyze the evolution of gene expression variance in outbred *Drosophila simulans* populations adapting to a new temperature regime to infer the genetic architecture of adaptive gene expression evolution. We suggest that the adaptive gene expression evolution is better explained by a polygenic architecture.

## Introduction

It is widely accepted that most complex traits have a polygenic basis (Ayroles et al. 2009; Boyle et al. 2017; Liu et al. 2019). Nevertheless, it is difficult to predict which or even how many of these loci are responding to selection when a population is exposed to a new selection regime (termed the “adaptive architecture” Barghi et al. 2020). Characterizing the adaptive architecture by mapping selected loci is not easy, in particular when more than a handful of genes are involved. To circumvent this problem, we introduce an approach, analogous to the Castle–Wright estimator (Castle 1921), to infer the complexity of the adaptive architecture (i.e. simple with few contributing loci or complex with a polygenic basis). We propose to study the evolution of phenotypic variance, which may provide some insights into the key parameters of the adaptive architecture.

The phenotypic variance of a quantitative trait is a key determinant for its response to selection. It can be decomposed into genetic and environmental components (Falconer and Mackay 1996). Over the past years, mathematical models have been developed to describe the expected genetic variance of a quantitative trait under selection and its maintenance in evolving populations (Kimura and Crow 1964; Bulmer 1972; Turelli 1984; Chevalet 1994). For infinitely large populations and traits controlled by many independent loci with infinitesimal effect, changes in trait optimum are not expected to affect the phenotypic variance (Lande 1976). A much more complex picture is expected when the effect sizes are not equal, the population size is finite, or the traits have a simpler genetic basis (Barton and Turelli 1987; Keightley and Hill 1989; Barton and Keightley 2002; Jain and Stephan 2015; Franssen et al. 2017; Hayward and Sella 2022). For instance, for traits with oligogenic architectures (typically <10 contributing loci), the genetic variance could drop dramatically during adaptation, while with polygenic architectures (≥10 contributing loci), only minor effects on the variance are expected (Jain and Stephan 2015; Barton et al. 2017; Franssen et al. 2017). These insights suggest that a time-resolved analysis of phenotypic variance has the potential to shed light onto the complexity of the underlying adaptive architecture.

Despite its potential importance for the understanding of adaptation, we are faced with the situation that few empirical data are available for the evolution of phenotypic variance. The use of natural populations to study changes in phenotypes, and even more so phenotypic variances, is limited as the environmental heterogeneity cannot be controlled and common garden experiments (CGE) are required to study the phenotypic variance, which is not feasible for many species. A complementary approach to study the evolution of phenotypic variance in natural populations is experimental evolution (Kawecki et al. 2012). With replicated populations starting from the same founders and evolving under tightly controlled environmental conditions, experimental evolution provides the opportunity to study the evolution of phenotypic variance.

Most experimental evolution studies in sexual populations focused on the evolution of phenotypic means, rather than variance (e.g. Chippindale et al. (1996), Burke et al. (2010), Mallard et al. (2018), and Jakšić et al. (2020)). A notable exception is a study which applied fluctuating, stabilizing, and disruptive selection to a small number of wing shape–related traits (Pélabon et al. 2010). Other studies tracked the variance evolution of behavioral and cranial traits under directional selection in mice (Careau et al. 2015; Penna et al. 2017). A challenge common to empirical studies on phenotypic variance in outbred populations is the partitioning of genetic and environmental variance.

Instead of looking at a preselected subset of phenotypes which may cover less variation in genetic architecture, we will focus on gene expression, a set of molecular phenotypes, which can be easily quantified as microarrays, and more recently, RNA-seq has become available. Importantly, the expression levels of genes exhibit the same properties (e.g. continuality and normality) as other complex quantitative traits (Mackay et al. 2009). Thus, gene expression has also been widely employed to study the adaptation of locally adapted populations (Romero et al. 2012; Sork 2017; Signor and Nuzhdin 2018) or ancestral and evolved populations in the context of experimental evolution (Lenski et al. 1994; Ferea et al. 1999; Huang and Agrawal 2016; Mallard et al. 2018).

In this study, we performed forward simulations that not only match essential design features of typical experimental evolution studies but also incorporate realistic parameters of the genetic architecture. We recapitulate the classic results that even a moderately polygenic architecture (i.e. 25 contributing loci) is associated with a high stability of the phenotypic variance of selected traits across different phases of adaptation and evaluate the predictive performance under different scenarios. Applying this insight to a recently published data set (Lai and Schlötterer 2022), we show that for putatively selected genes (differentially expressed [DE] genes), their average changes in expression variance were indistinguishable from genes without changes in mean expression. We propose that this pattern reflects a polygenic basis of adaptive gene expression evolution.

## Results

### Simulating Variance Evolution of Expression Traits with Distinct Genetic Architectures

**Fig. 1.**
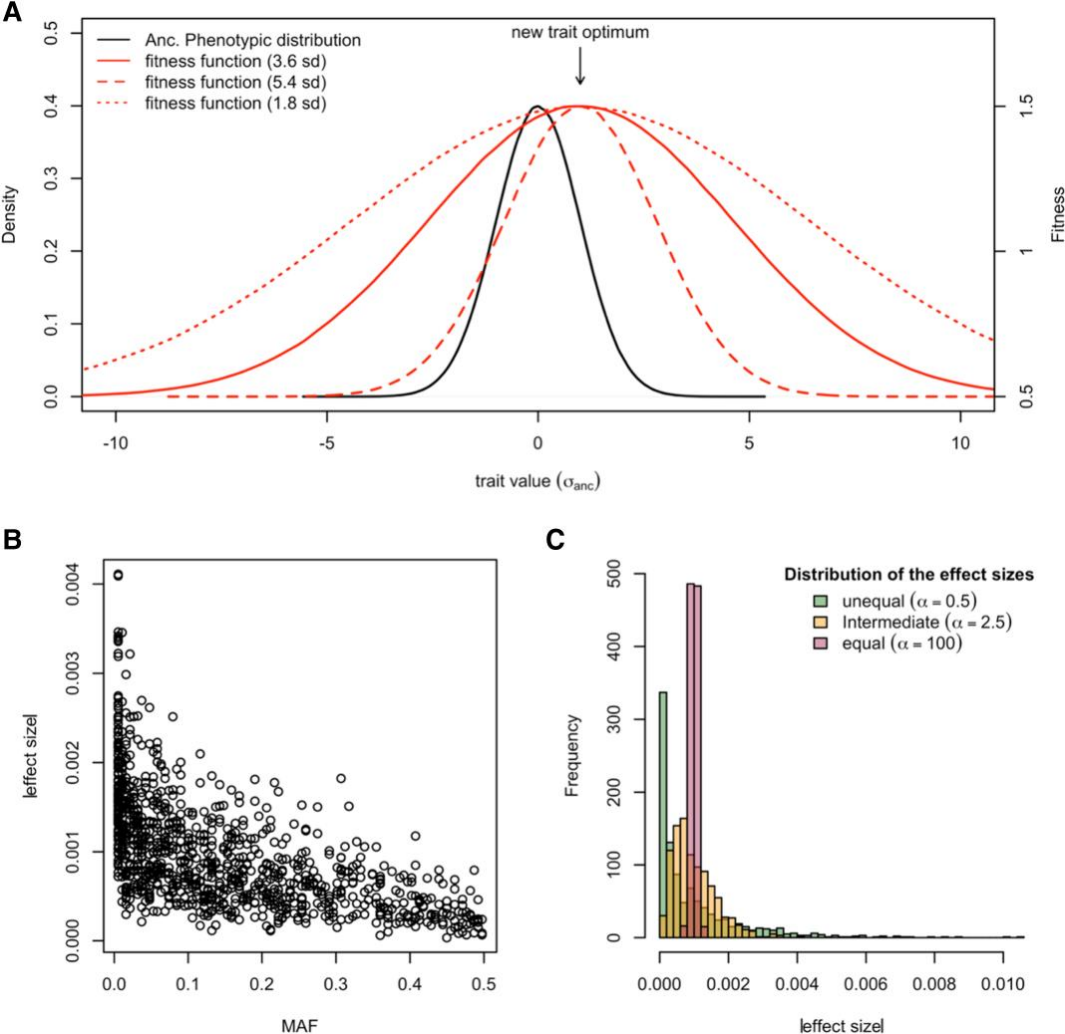
Simulating polygenic adaptation to a shift in trait optimum with different parameter combinations. a) For the computer simulations, we consider a quantitative trait experiencing a sudden shift in trait optimum under stabilizing selection. Three underlying fitness functions are shown. The new trait optimum is shifted from the ancestral trait mean by one/three standard deviation of the ancestral trait distribution. The strength of stabilizing selection is modified by changing the variance of the fitness function: 1.8, 3.6, and 5.4 standard deviations of the ancestral trait distribution. b) The negative correlation between the allele frequencies and the effect sizes (*r* = −0.7). We consider such negative correlation when assigning the effect sizes to variants underlying a simulated trait. c) The distribution of effect sizes of the contributing loci is determined by the shape parameter (*α*) of gamma sampling process (α = 0.5, 2.5, and 100).

The central idea of this study is that the genetic complexity of adaptive trait evolution can be inferred from the trajectory of the phenotypic variance during adaptation: the phenotypic variance remains relatively stable for traits with a polygenic architecture, while it changes across generations for traits with a oligogenic architecture. Hence, as the first step of this study, we explored to what extent these theoretical predictions can be generalized to expression traits obtained from a typical experimental evolution setting by considering a broad parameter space and accounting for linkage. Using population genetic parameters (e.g. number of starting haplotypes and effective population size) estimated from the evolution experiment (Barghi et al. 2019), we simulated the independent evolution of 1,000 neutral and 1,000 selected expression traits. To account for experimental and environmental noise, we assigned heritabilities of expression traits based on an empirical distribution obtained from a *Drosophila melanogaster* population (Ayroles et al. 2009). The simulated selection regime consists of a mild/distant shift in trait optimum with weak/intermediate/strong stabilizing selection (Fig. 1a and supplementary fig. S1, Supplementary Material online) following the parameterization of a recent simulation study (Hayward and Sella 2022). We assumed additivity and a negative correlation between the ancestral allele frequency and the effect size of contributing loci (Otte et al. 2021) (Fig. 1b). With three different distributions of effect size (Fig. 1c), we investigated how the number of contributing loci affects the evolution of gene expression variance with and without selection.

**Fig. 2.**
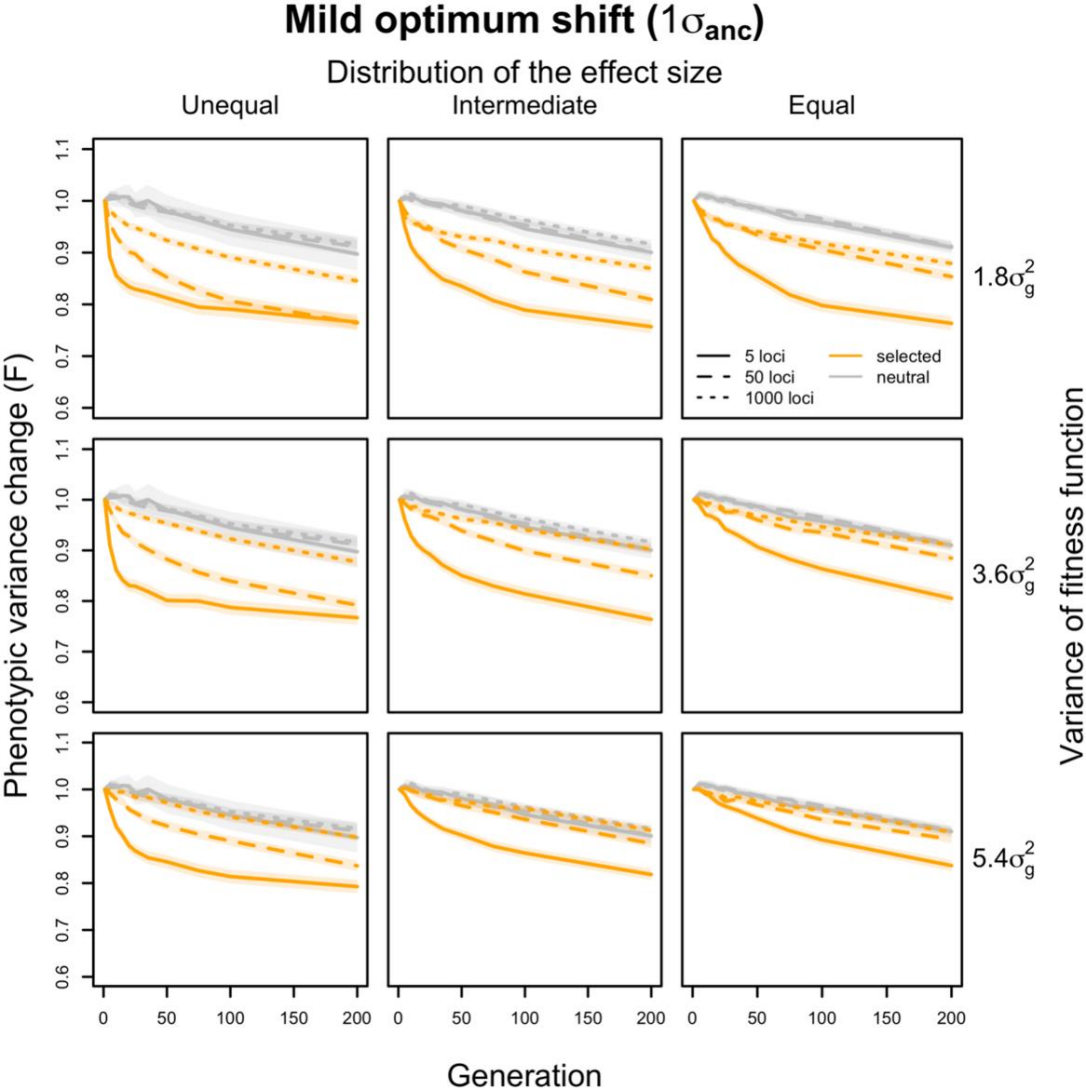
The trajectory of changes in phenotypic variance during adaptation to a mild optimum shift. The changes in phenotypic variance within 200 generations adapting to a moderate optimum shift are compared with the changes under neutrality on the *y* axis. The average variance changes (*F*) of 1,000 simulated traits (1,000 runs of simulation) are calculated as the ratio of phenotypic variance between each evolved time point (generation *x*) and the ancestral state ($\sigma_{x}^{2}/\sigma_{1}^{2}$). The translucent band indicates the 95% confidence interval for 1,000 simulated traits. The simulations cover traits controlled by varying numbers of loci underlying the adaptation with three different distributions of effect sizes (columns) under different strengths of stabilizing selection (rows). Only traits with the most (dotted lines, 1,000 loci), intermediate (dash lines, 50 loci), and the least (solid lines, 5 loci) polygenic architectures are shown. In all scenarios, the variance of the trait decreases drastically when the adaptation is controlled by a small number of loci (solid lines; 5 loci). In contrast, for traits with extremely polygenic basis, the phenotypic variance decreases less over time (dotted lines).

We monitored the change in phenotypic variance over 100 generations, which was sufficient to reach the trait optimum for most parameter combinations (supplementary fig. S2, Supplementary Material online). We compared the change in variance relative to the start of the experiment in populations with and without selection. First, we studied a mild (one standard deviation of the ancestral phenotypic distribution) shift in trait optimum. As expected for a founder population derived from a substantially larger natural population, we find that even under neutrality, the phenotypic variance does not remain constant but gradually decreases during 100 generations of experimental evolution (Fig. 2). This pattern is unaffected by the genetic architecture (both number of loci and effect size distribution) of the neutral traits. We explain this loss of variance by the fixation of variants segregating in the founder population and the fact that we did not simulate new mutations, as they do not contribute much to gene expression evolution in such short time scales (Rifkin et al. 2005; Burke et al. 2010). Although our simulations used moderate population sizes, they nicely recapitulate the patterns described for populations without drift (Jain and Stephan 2015; Barton et al. 2017). A pronounced drop in phenotypic variance is observed when a trait is approaching a new optimum with few contributing loci (Fig. 2). When more loci (with smaller effects) are contributing to the selected phenotype, the difference to neutrality becomes very small (Fig. 2). In addition to the number of contributing loci, the heterogeneity in effect size among loci and the shape of the fitness function have a major impact. The larger the difference in effect size is, the more pronounced was the influence of the number of contributing loci (Fig. 2). The opposite effect was seen for the width of the fitness function—a wider fitness function decreased the influence of the number of contributing loci (Fig. 2). Importantly, these patterns were not affected by the duration of the experiment—qualitatively identical patterns were seen at different time points until generation 200.

For a more distant trait optimum (three standard deviations of the ancestral phenotypic distribution away from the ancestral value), we noticed some interesting dynamics that were not apparent for a closer trait optimum (supplementary fig. S3, Supplementary Material online). The most striking one was the temporal heterogeneity of the phenotypic variance when few loci of unequal effects are contributing. During the early stage of adaptation, the variance increased and dropped later below the variance in the founder population. With an increasing number of contributing loci, this pattern disappeared and closely matched the neutral case (supplementary fig. S3, Supplementary Material online).

**Fig. 3.**
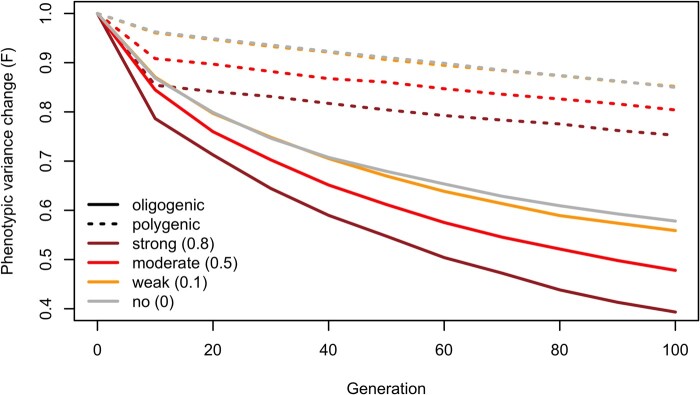
The variance dynamics of modularly regulated traits during adaptation. We monitored the average variance changes relative to the ancestral state of 2,000 simulated traits under modular regulation (100 modules of 20 expression traits) within 100 generations of adaptation. The number of contributing loci to the modules was varied to simulate oligogenic (solid lines) and polygenic (dash lines) architectures. The effect size of each contributing loci was drawn from a multivariate normal distribution. The covariance among expression traits was set to 0, 0.1, 0.5, and 0.8 for random, weak, moderate, and strong genetic correlation among the selected traits (indicated by different colors), respectively. In all cases, we recapitulated the pattern that oligogenic architecture of expression traits leads to a drastic drop in variance over time.

Overall, our simulations indicate that with a larger number of contributing loci, the variance fitted the neutral pattern better. Modifying dominance did not change the overall patterns (supplementary fig. S4, Supplementary Material online). Neither did a modular regulation of expression traits. We monitored the variance evolution in simulations with 100 modules of 20 coregulated traits and different numbers of contributing loci. Consistently, simple genetic architectures lead to a drastic drop in phenotypic variance (Fig. 3). The strength of coregulation has only a minor impact on the pattern (Fig. 3). The robust influence the number of contributing loci and their effect size distribution on the temporal phenotypic variance dynamics suggests that it should be possible to exploit this relationship to characterize the adaptive architecture.

### The Power to Infer the Adaptive Architecture from Trait Variance Dynamics

A potentially interesting application of the relationship between number of contributing loci and evolution of variance is the inference of the adaptive architecture of a given trait. For a single selected trait, it is possible to contrast the reduction in variance for this trait to the change in variance at neutral traits. We used an *F* test to identify a significantly higher loss of variance for focal traits than neutral ones (∼0.9 after 100 generations in our simulations). Because the number of contributing loci is not known, it is important to determine the lower bound for the number of contributing loci that produces a pattern of variance change that cannot be distinguished from neutrality (a more complex architecture will result in a pattern similar to neutrality). We inferred the lower bound by the largest number of loci resulting in a significant difference between a selected trait and the neutral expectation across all simulated parameter combinations. Nevertheless, the power of this approach depends strongly on the sample size. When the entire population (*N* = 300) is phenotyped for the focal trait, the power to rule out an adaptive architecture of <5 loci by significant decrease in variance of a selected trait is only 44%. This indicates the limited ability to distinguish polygenic from oligogenic architectures on a single trait basis. With a more realistic sample size of 20 individuals, the distinction is even more difficult such that we conclude that it is not possible to infer the adaptive architecture for a single expression trait (supplementary fig. S5, Supplementary Material online).

**Fig. 4.**
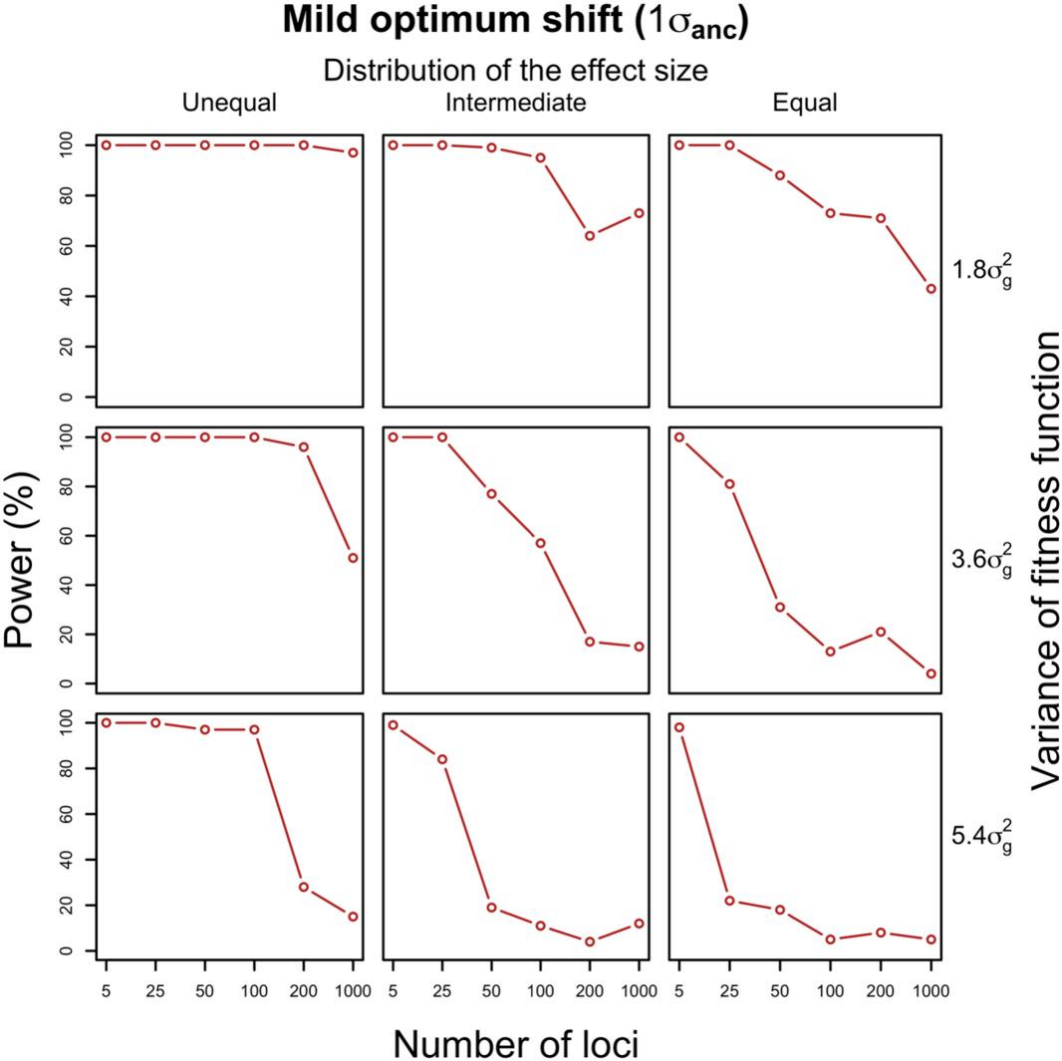
Power for detecting significant variance change between groups of selected and neutral traits. For each set of the 1,000 traits (1,000 runs of simulation) controlled by different numbers of loci (*x* axis) with varying effect sizes (columns) under each selection strength (rows), we calculated how often no difference in variance change is detected between 1,000 neutral and 1,000 selected traits after 100 generations (*y* axis). The same genetic architecture is assumed for all 1,000 selected traits with a sample size of 20. For each parameter combination, we have almost 100% power when all traits were controlled by few loci (five loci). It gradually increases with an increasing number of loci (red). A lack of significant difference indicates polygenic architecture of the adaptive traits.

Alternatively, it is possible to study multiple, presumably independent, selected phenotypes together. Assuming a similar genetic architecture of the selected traits, we showed that the distribution of variance changes of 1,000 selected traits with a simple genetic architecture (five loci) can be distinguished from neutral changes with a *t*-test. Even with a sample size of 20 individuals, as in our experimental data (see Materials and Methods), significant differences from neutral expectations can be detected for some parameters with a power close to 100% (Fig. 4 and supplementary fig. S6, Supplementary Material online). Because the distribution of variance changes between selected and neutral polygenic traits does not differ significantly, this suggests that oligogenic and polygenic architectures of a group of selected traits can be distinguished experimentally even with moderate sample sizes.

**Fig. 5.**
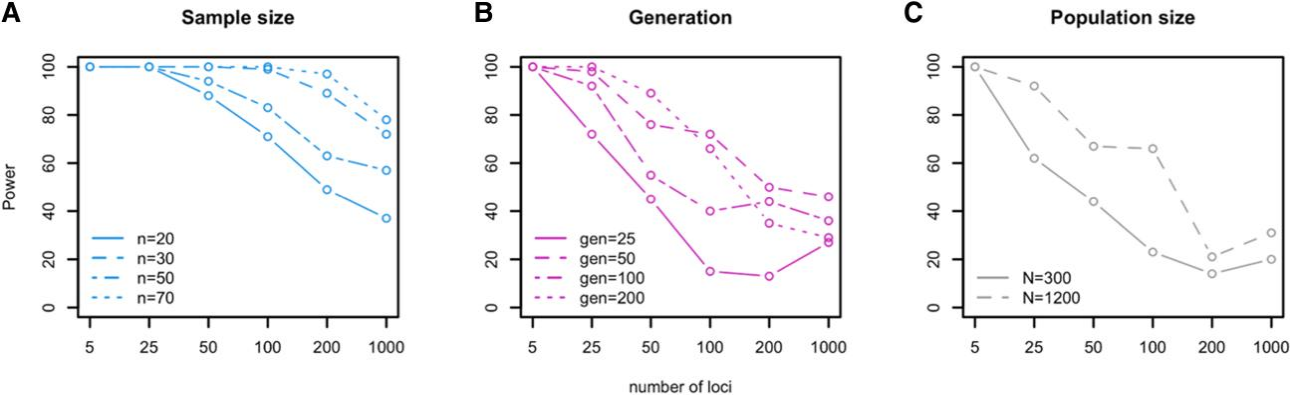
Power for detecting significant variance change between groups of selected and neutral traits under different a) sample sizes, b) generations, and c) population sizes. For each set of the 1,000 traits (1,000 runs of simulation) controlled by different numbers of loci (*x* axis) under different experimental conditions, we calculated how often a difference in variance change is detected between 1,000 neutral and 1,000 selected traits (*y* axis). The same genetic architecture is assumed for all 1,000 selected traits. a) The power gradually increases with larger sample sizes. b) Given the same number of loci, a higher power is obtained when the experiment continues for more generations. c) Experiments with larger population size (*N* = 1200) have a higher power than those with a smaller population size (*N* = 300). Overall, larger and longer evolution experiments with more phenotyped samples provide more power to reject simpler adaptive architectures and thus provide more confidence in a highly polygenic architecture when we observed no significant difference in variance.

In the simulations, we fixed most experimental parameters such as population sizes, generation, and sample sizes to reflect the experimental design of typical experimental evolution studies. Nevertheless, for future experimental evolution studies, it is important to understand how the experimental design affects the power. We performed additional simulations to explore the impact of sample size, number of generations, and population size. As expected, the power increases with larger sample size, allowing to discriminate architectures with a larger number of contributing loci (Fig. 5a). Later generations are also more informative than earlier generations (i.e. higher power to reject a larger number of loci) (Fig. 5b). Larger population sizes could also improve the determination of polygenicity, as we find an increase in power for a population size of 1,200 compared with 300 (Fig. 5c). In summary, larger and longer evolution experiments with larger sample size are superior. They provide a better discrimination of genetic architectures with a larger number of contributing loci, i.e. a better distinction between oligogenic and polygenic architectures.

### Empirical Data on Evolution of Gene Expression Variance Suggests the Polygenic Basis

**Fig. 6.**
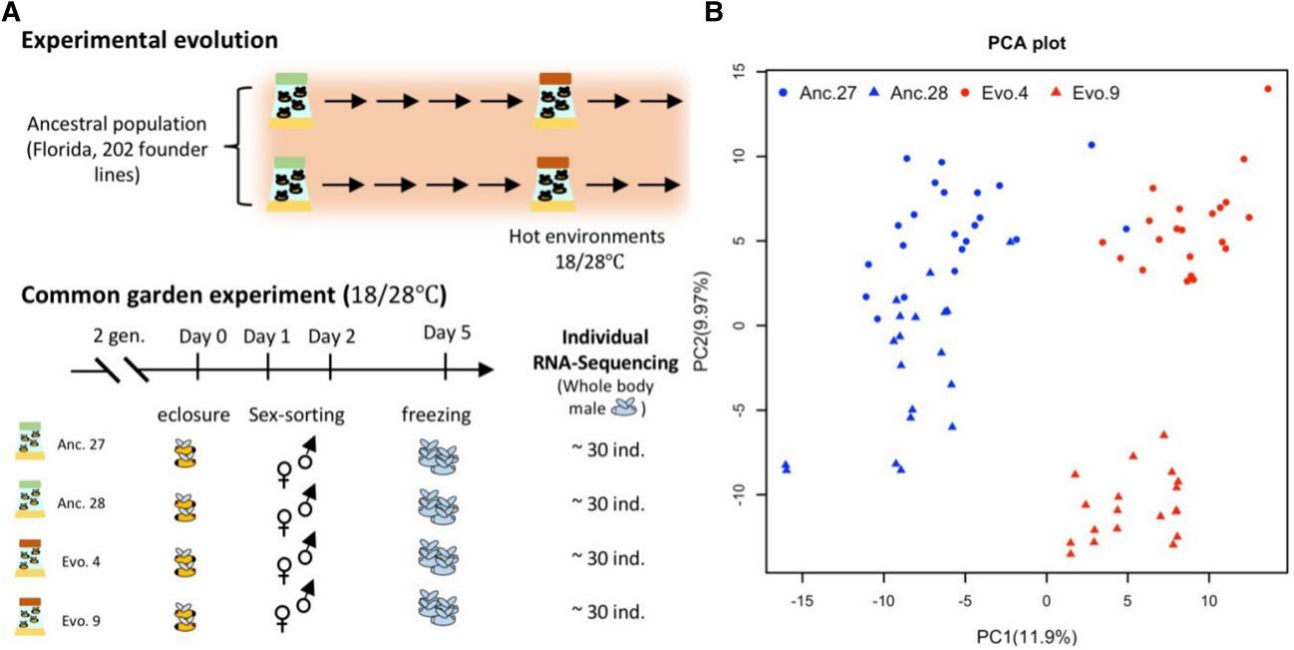
Schematic overview of the experimental procedures (a) and the divergence in gene expression during experimental evolution (b). a) Experimental evolution: starting from 1 common founder population, 2 replicate populations evolved for 100 generations in a hot laboratory environment fluctuating between 18 and 28 °C. CGE: after 100 generations, the 2 evolved replicate populations were maintained together with the reconstituted ancestral population for 2 generations in a hot laboratory environment fluctuating between 18 and 28 °C. After this common garden procedure, males from each population were subjected to RNA-seq. b) PCA of the transcriptomic profiles of individuals from the ancestral population (Anc.) and the hot-evolved population (Evo.). Circles indicate individuals of the first replicate (Anc. No. 27 and Evo. No. 4). Triangles represent individuals of the second replicate (Anc. No. 28 and Evo. No. 9). The two replicates were made with two different batches of library cards for RNA-seq library preparation. The impact of the library card batch can be seen from the separation of the ancestral replicates which were reconstituted from the same isofemale lines.

As a case study, we investigated the evolution of gene expression variance in replicated populations evolving in a new hot temperature regime and inferred the adaptive architecture of gene expression evolution. The evolved populations were derived from the same ancestral population but evolved independently for more than 100 generations in a novel temperature regime with daily temperature fluctuations between 18 and 28 °C (Fig. 6a). Rather than relying on pooled samples which only allow mean estimates, we quantified gene expression of 19 to 22 individuals from reconstituted ancestral populations (F0) and 2 evolved populations (F103) in a common garden setup (Lai and Schlötterer 2022). Principal component analysis (PCA) indicated that 11.9% of the variation in gene expression can be explained by the first PC which separates evolved and ancestral populations, reflecting clear expression changes in response to the adaptation of novel hot temperature regime (Fig. 6b). The means and variances of the expression of each gene were estimated and compared between the two reconstituted ancestral populations and the two evolved populations separately (see Materials and Methods). Due to the usage of different lot numbers for the RNA-seq library preparation (supplementary table S1, Supplementary Material online), we only contrasted ancestral and evolved samples generated with the same lot number (see Materials and Methods) to avoid any unnecessary confounding effects.

**Fig. 7.**
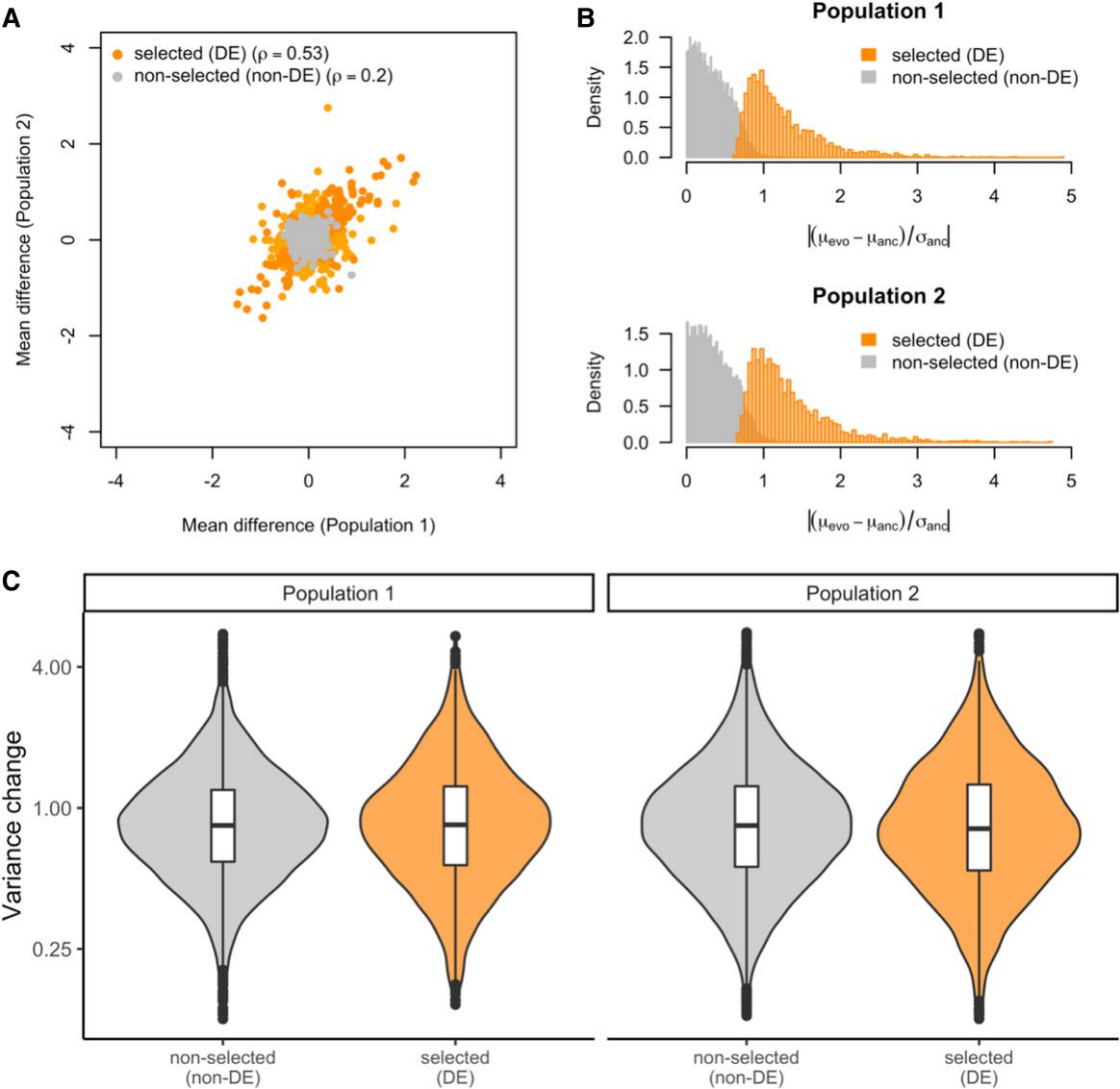
Evolution of phenotypic mean and variance over 100 generations of adaptation in empirical data. a) The evolution of gene expression means during adaptation in the two replicates. For the genes with significant changes (DE), the changes are correlated between replicates (Spearman's rho = 0.53). For the genes without significant changes (non-DE), the correlation between replicates is much lower (Spearman's rho = 0.2). b) The evolution of gene expression means scaled by the ancestral variation. For the DE genes, the median change is around one standard deviation of the ancestral expression value, suggesting mild shift in trait optimum in the novel environment. For the non-DE genes, the changes in expression are mostly negligible. The same pattern is seen in the second replicate. c) The change in expression variance during adaptation for DE and non-DE genes. In both replicates, the distribution of variance changes is indistinguishable between DE genes and non-DE genes (*t*-test, *P* > 0.05 for both replicates). The almost identical pattern is observed when the BCV (see Materials and Methods) is used to estimate the expression variance (supplementary fig. S4, Supplementary Material online).

As reported previously (Lai and Schlötterer 2022), we identified 2,775 genes in the first population and 2,677 genes in the second population which significantly changed mean expression in the evolved flies (false discovery rate [FDR] < 0.05) and significant parallel gene expression evolution between 2 populations (Fig. 7a). The concordance of both populations suggests that most of the altered expression means are mainly driven by selection, rather than by drift. In this study, we scaled the gene expression change with the standard deviation in the ancestral population to approximate the selection strength on each gene. The differentially expressed genes in both populations showed a broad distribution of expression change, but the averaged mean expression changed by one standard deviation (Fig. 7b), which is unlikely to be reached by drift based on our simulations (<1% of the neutral traits drifted by one standard deviation of the ancestral phenotypic variance). Given that these evolved populations have been suggested to reach trait optimum (Christodoulaki et al. 2022), the observed mean shift in expression of about one standard deviation corresponds to a mild shift in trait optimum in our computer simulations.

In both replicates, the expression variance of putative neutral (non-DE) genes dropped to 84% of the ancestral variance, which is quite close to the neutral cases in our simulations. While it would be desirable to test each putatively selected (DE) gene independently, the moderate sample size (∼20 per population) does not provide sufficient power to do so (supplementary fig. S8, Supplementary Material online). Rather, we considered all putatively selected (DE) genes jointly and compared their variance changes with the ones of putatively neutral (non-DE) genes to characterize the genetic basis of the expression evolution of the selected genes as a group. Our power analysis suggests a decent power for this test (Fig. 4). Remarkably, we find that the changes in variance of putatively selected genes with significant mean expression changes are indistinguishable from the genes that do not change their mean expression (*t*-test, *P*-value > 0.05; Fig. 7c). The magnitude of gene expression variance changes in both sets of genes (median *F*-value = 0.85 and 0.84 for DE and non-DE genes, respectively, in population 1 and 0.82 and 0.84 in population 2) is quite close to the neutral conditions in our computer simulations. This suggests that selection on mean expression did not significantly change the expression variance during adaptation, and thus, the expression of most putatively adaptive genes has polygenic basis (i.e. more than five contributing loci).

## Discussion

Population genetics has a long tradition of characterizing adaptation based on the genomic signature of selected loci (Nielsen 2005). Nevertheless, for selected phenotypes with a polygenic architecture, the contribution of individual loci to phenotypic change may be too subtle to be detected with classic population genetic methods (Pritchard et al. 2010). Although identification of many small-effect loci is challenging, it may be possible to determine the number of contributing loci based on the dynamics of phenotypic variance.

Inspired by the Castle–Wright estimator (Castle 1921) that estimates the number of loci contributing to a quantitative trait from the phenotypic variance of the F2, we propose that the temporal heterogeneity of the phenotypic variance can potentially be used to infer the number of loci contributing to the adaptive response of a phenotype as well as other parameters of the adaptive architecture. Reasoning that experimental evolution is probably the best approach to obtain phenotypic time series, we performed computer simulations specifically tailored to typical experimental evolution studies with *Drosophila*. We demonstrated that, in an experimental evolution setup, the temporal dynamics of the phenotypic variance can be used to estimate the number of contributing loci and other parameters such as the distribution of effect size.

One potential limitation of our analysis is that we assumed neutrality of non-DE genes; it may, however, be possible that they are also subject to stabilizing selection but without a shift in trait optimum. In this case, the same change in variance is expected for both groups of traits—even under an oligogenic architecture, as shown by computer simulations (supplementary fig. S7a and b and supplementary information, Supplementary Material online). If stabilizing selection is also operating on non-DE genes, the change in variance should be correlated between replicate populations because each trait experiences the same strength of stabilizing selection (each trait has the same heritability in both replicates, but it differs between traits). This prediction was confirmed in our computer simulations (supplementary fig. S7c, Supplementary Material online). Our empirical data indicate, however, that the correlation of variance change differs between DE and non-DE genes. The change in variance exhibited a stronger correlation between the two evolution replicates of the DE genes (*r* = 0.07) than between the two replicates of the non-DE genes (*r* = 0.02). This implies that stabilizing selection had only a weak or no effect on the variance of non-DE genes. This difference in correlation is highly significant when we compared the correlation coefficient of DE genes with the same number of randomly drawn non-DE genes (*P* < 0.01; supplementary fig. S7d, Supplementary Material online). Given that the distribution of correlation coefficients for non-DE genes is very close to the expectations under neutrality from computer simulations, we think that the behavior is better approximated by neutrality for most of the non-DE genes, rather than assuming similar levels of stabilizing selection for both classes of genes. It is not clear, however, whether our results reflect a much broader fitness function determining the evolution of non-DE genes per se or the simple laboratory environment relaxes selection on non-DE genes.

Given the limited power to infer the number of contributing loci for each expression phenotype (supplementary fig. S5, Supplementary Material online), we grouped all putatively selected expression traits and limited our inference of the adaptive architecture to these genes jointly. This approach makes the implicit assumption that all expression traits in this group are independent of each other and have similar level of complexity in their adaptive architectures. Thus, the joint inference could potentially compromise the detection of a minor subset of genes whose expression evolution is under simple genetic control.

Because we could only analyze phenotypic data from 2 time points and the founder population and replicate populations evolved for 103 generations, we were not able to obtain a more quantitative estimate of the number of contributing loci, in particular as other parameters of the adaptive architecture are not known and need to be coestimated. In addition, with only two time points, for a few parameter combinations, an oligogenic response can also result in a similar phenotypic variance change as a polygenic one (supplementary figs. S3 and S6, Supplementary Material online) but with a much higher parallel response of genomic markers (supplementary fig. S8, Supplementary Material online). This can be seen in an intuitive case when a single/few major effect allele(s) starts at a low frequency and sweeps up to fixation (Yoo et al. 1980). Because the genomic signature in the same experiment uncovered a highly heterogeneous selection response (Barghi et al. 2019), we can exclude the unlikely explanation of an oligogenic architecture of expression evolution in our empirical study. Hence, not only more time points describing the phenotypic trajectory but also genomic data could improve the inference of the adaptive architecture in experimental evolution studies.

The extension of the concept proposed in this study to natural populations could face several challenges that warrant extra caution and further investigation. First, the abovementioned challenge of sample size would be more pronounced in a natural setup. Second, phenotypic time series over evolutionary relevant time scales are costly (but see Clutton-Brock et al. (2004) for an example of time series in the wild), and third, the distinction of environmental heterogeneity from genetic changes is considerably more challenging than under controlled laboratory conditions.

## Materials and Methods

### Computer Simulations

#### Software and Fixed Parameters

We performed forward simulations with MimicrEE2 (Vlachos and Kofler 2018) using the qff mode to illustrate the influence of the genetic architecture on the evolution of phenotypic variance during the adaptation to a new trait optimum (Fig. 1a and supplementary fig. S1, Supplementary Material online). With 189 founder haplotypes (Barghi et al. 2019), we simulated quantitative gene expression traits in a population with an effective population size of 300 (reflecting the estimated effective population size in an experimentally evolving population (Barghi et al. 2019)). We used the empirical linkage map from *Drosophila simulans* (Howie et al. 2019) to account for linkage. For each trait, we assume an additive model and a negative correlation (*r* = −0.7, reflecting the observed relationship from Otte et al. (2021)) between the effect size and starting frequency (Fig. 1b). We used the *correlate()* function implemented in “fabricatr” R package (Blair et al. 2019) to generate the effect sizes. We do not simulate de novo mutations as they are not contributing to adaptation on this short time scale (Rifkin et al. 2005; Burke et al. 2010).

#### Parameterization of Different Genetic Architectures

The central idea of this study is that the genetic complexity of adaptive trait evolution can be inferred from the trajectory of the phenotypic variance during adaptation. Hence, we varied the genetic architecture of simulated expression traits in two aspects. First, we varied the number of contributing loci (*M* = 5, 25, 50, 100, 200, and 1,000). Second, we varied the distribution of the effect sizes of the loci controlling a trait by changing the shape parameter of gamma sampling process (shape = 0.5, 2.5, and 100; Fig. 1c). The mean of the effect sizes from three different shape parameters was standardized to 1/M. As the focus of our study is on expression traits, we used the distribution of heritabilities obtained from empirical gene expression data (Ayroles et al. 2009) to assign the environmental/technical variance to a simulated trait (i.e. for each simulated trait, its heritability and associated environmental/technical variance are sampled from the empirical distribution). We note that the heritabilities in Ayroles et al. (2009) may be overestimated because of the use of pooled sample measurements, but we are still lacking gene expression heritability estimates based on individual sequencing in the fruit flies field.

#### Selection Regimes

To simulate stabilizing selection with trait optimum shift, we provided the Gaussian fitness functions with mean of $\bar{X_{\mathrm{anc}.}}+a\sqrt{V_{\mathrm{anc}.}}$ and standard deviation of $\;b\sqrt{V_{\mathrm{anc}.}}$, where $\bar{X_{\mathrm{anc}.}}$ is the ancestral phenotypic mean and $V_{\mathrm{anc}.}$ is the ancestral genetic variance (Fig. 1a). The inclusion of the parameter *V*_anc._ scales the selection strength, which allows the comparison across simulation runs from the entire parameter space. Parameter “*a*” determines the distance of optimum shift, which is set to one (similar to the empirical case; Fig. 7b) or three (following Hayward and Sella (2022)). Parameter “b” indicates the phenotypic constraint would be at trait optimum. The value 3.6 for parameter “b” was taken from Hayward and Sella (2022). In this study, we increase and decrease it by 50% to explore its impact (1.8 or 5.4). For the neutral case, we assumed no variation in fitness among all individuals.

#### Replication and Simulated Data Analysis

In total, 162 parameter combinations were simulated (6 different numbers of loci × 3 distributions of effect sizes × 3 different widths of fitness function × 3 selection regimes). For each scenario, we performed 1,000 independent runs of simulation to represent 1,000 different traits. Each trait was affected by a different set of loci and evolved independently.

We compared the phenotypic variance of selected and neutral traits for each genetic architecture. For each trait under each scenario, the phenotypic variance was estimated at different generations and compared with the ancestral phenotypic variance at generation 1 to illustrate the dynamic of phenotypic variance during the evolution ($F=\sigma_{x}^{2}/\sigma_{1}^{2})$, where *x* stands for the number of generations. We note that we do not assume that the ancestral population has reached an equilibrium because the ancestral population in a typical experimental evolution study is often phenotyped in the new environment.

#### Additional Parameter Changes for Different Experimental Evolution Setup

In addition, we evaluated how different experimental designs (e.g. different population sizes in the experiment) affect the ability to discriminate between different adaptive architectures. With all other parameters fixed, we increased the population size to 1,200 and simulated the evolution of traits controlled by a different number of loci (*M* = 5, 25, 50, 100, 200, and 1,000) with varying effect sizes (shape parameter of gamma sampling process = 2.5) using a Gaussian fitness function with a mean of $\bar{X_{\mathrm{anc}.}}+\sqrt{V_{\mathrm{anc}.}}$ and a standard deviation of $\;3.6\sqrt{V_{\mathrm{anc}.}}$.

#### Simulation Considering Modularity

Given the software limitation of MimicrEE2, we extend our simulation to account for modular regulation of gene expression using SLiM (Haller and Messer 2023). This simulation burnt in with 20,000 generations of neutral evolution of 5,000 weakly linked loci (*r* = 0.001) in an ancestral population sizing 10,000 (Ne = 10,000) to reach mutation–drift–linkage balance. After the burn-in phase, we sampled 300 individuals from the ancestral population to seed the simulation experiment, mirroring the experimental evolution setup. We simulated the evolution of 100 independent expression modules of 20 genes per module, yielding a total of 2,000 expression traits. The genetic loci regulating genes in one module were assumed to have no effect on the genes in the other modules. To vary the genetic architecture of each expression module, either all or 5% of all segregating variants were assigned effect sizes sampling from a multivariate normal distribution of 20 variates (trait), standing for highly polygenic or oligogenic basis of the traits. We simulated random, weak, moderate, and strong positive modularity with trait covariance of 0, 0.1, 0.5, and 0.8 correspondingly. As for the selection regime, we simulate the same stabilizing selection with trait optimum shift of one standard deviation of the ancestral trait. For each module, the high dimensional fitness landscape of 20 traits follows a multivariate Gaussian fitness function:


$$\omega =\exp\left(-\frac{1}{2}\overset{20}{\underset{i=1}{\sum }}\frac{{\left(z_{i}-z_{0}\right)}^{2}}{2V_{s}}\right)$$

where $z_{i}$ is the observed phenotypic value for a trait *i*; $z_{0}$ is the optimum phenotypic value, which is one standard deviation away of the ancestral value ($\bar{X_{\mathrm{anc}.}}+\sqrt{V_{\mathrm{anc}.}}$); and $V_{s}$ is the variance of the fitness profile ($3.6\sqrt{V_{\mathrm{anc}.}}$). Two genetic architectures (polygenic and oligogenic), four scenarios of modular effects lead to a total of eight unique parameter setups. Each was repeated 100 times to get 100 expression modules (2,000 expression traits) per scenario. Similarly, we compared the phenotypic variance of the traits for each genetic architecture. We further extended the simulation to the scenario by assuming connected modules (i.e. a gene shares the genetic architecture within and between modules, but the level of genetic correlation differs). This scenario yielded qualitatively similar results (supplementary fig. S9, Supplementary Material online).

### Evaluating the Ability to Distinguish Adaptive Architectures Based on Trait Variance Evolution

We propose that the temporal dynamics of the phenotypic variance of selected traits can be exploited to characterize the genetic basis of adaptation. This concept could be applied either to a single trait under selection or to a group of selected traits. Depending on whether a single trait or a group of traits is studied, different research objectives can be pursued. Generally speaking, a more complex architecture is expected to result in a variance pattern that resembles neutral evolution (i.e. no change in variance). A lower bound for the number of contributing loci can be inferred by asking for the maximum number of contributing loci causing a variance change during evolution, which significantly deviates from neutral expectations. With increasing uncertainty about the key parameters, the precision of this approach is reduced. Given that many parameters are typically not known, we performed computer simulations for different numbers of loci with a distribution of effect sizes and asked for each focal parameter combination whether a significant deviation from neutral expectations was observed. The number of simulation runs, which deviate from neutral expectations for *x* loci, is used as power estimate to reject the null hypothesis of no difference to the variance under neutrality under a given architecture and sample size. In other words, it reflects the confidence to exclude an adaptive architecture with *x* or fewer contributing loci when we observed no significant difference in variance.

#### Power Estimates for the Analysis of a Single Trait

For each simulated trait under different genetic controls, we tested whether the variance of this trait changed more than expected under neutrality (*F* of 0.9 according to the neutral simulations) after 100 generations of selection. First, we assumed that all simulated 300 individuals were phenotyped at both time points. For the 1,000 traits with a given genetic architecture (*x* contributing loci) under each selection scenario, we calculated the power to reject the null hypothesis of an adaptive architecture of no difference to the variance under neutrality under a given architecture and sample size. Since it is not possible to phenotype all individuals, we also investigated whether and how a reduced sample size compromises the power to reject an adaptive architecture of a single trait based on the change in variance. We randomly selected 20 individuals from each simulation run to estimate the variance and tested whether the magnitude of variance change for a given trait under selection is significantly different from the neutral expectation.

#### Power Estimates for the Analysis on a Group of Traits

We assessed the power to infer the adaptive architecture for a group of selected traits under the assumption that most traits of interest have similar level of complexity. In this case, a test on the average variance changes (*F*) between a group of selected traits and another group of neutral traits is required. As described, we simulated 1,000 independent selected and 1,000 neutral traits for each genetic architecture and selection regime and estimated the variance before and after 100 generations of evolution for the selected and neutral traits. For each trait, we measured the phenotypic variance for a sample of 20 individuals and we performed a *t*-test to compare the distribution of variance change (*F*) between selected and neutral traits. The power was determined by 100 iterations of sampling 20 individuals from a simulated population of 300 individuals.

We further evaluated how different experimental designs affect the power to detect simpler adaptive architectures. We used the data from the scenario with shape parameter 2.5 and Gaussian fitness functions with mean of $\bar{X_{\mathrm{anc}.}}+\sqrt{V_{\mathrm{anc}.}}$ and standard deviation of $\;3.6\sqrt{V_{\mathrm{anc}.}}$. With this data set, we calculated the power for different sample sizes (*n* = 20, 30, 50, 70), generation times (gen = 25, 50, 100, 200), and population sizes (*N* = 300 and 1,200).

### Experimental Evolution

The setup of populations and evolution experiment has been described by Barghi et al. (2019), Hsu et al. (2019, 2020), Jakšić et al. (2020), and Lai and Schlötterer (2022). Briefly, 10 outbred *D. simulans* populations seeded from 202 isofemale lines were exposed to a laboratory experiment at 28/18 °C with 12 h light/12 h dark photoperiod for more than 100 generations. Each replicate consisted of 1,000 to 1,250 adults for each generation.

### CGE

The collection of samples from the evolution experiment for RNA-seq was preceded by two generations of common garden (CGE). The CGE was performed at generation 103 of the evolution in the hot environment, and this CGE has been described in Hsu et al. (2019), Hsu et al. (2020), Jakšić et al. (2020), and Lai and Schlötterer (2022). In brief, an ancestral population was reconstituted by pooling 5 mated females from 184 founder isofemale lines (Nouhaud et al. 2016). No significant allele frequency differences are expected between the reconstituted ancestral populations and the original ancestral populations initiating the experiment (Nouhaud et al. 2016). Furthermore, we do not anticipate that deleterious alleles acquired during the maintenance of the isofemale lines had a major impact on the phenotypic variance in the reconstituted ancestral population. The reason is that novel deleterious mutations occurring during the maintenance of the isofemale lines are present in a single isofemale line only. Given the large number of isofemale lines (184), such deleterious alleles occur in a low frequency in the reconstituted population with a small influence on the phenotypic variance (Walsh and Lynch 2018). Furthermore, most of these deleterious alleles are present in heterozygous individuals and masked because deleterious alleles tend to be recessive (Charlesworth and Charlesworth 2010). As described previously (Lai and Schlötterer 2022), 2 replicates of the reconstituted ancestral population and 2 independently evolved populations at generation 103 were reared for 2 generations with controlled egg density (400 eggs/bottle) at the same temperature regime as in the evolution experiment. Freshly eclosed flies were transferred onto new food for mating. Sexes were separated under CO_2_ anesthesia at day 3 after eclosure and left to recover from CO_2_ for two days, and at the age of five days, whole-body mated flies of each sex were snap-frozen at 2 PM in liquid nitrogen and stored at −80 °C until RNA extraction. More than 30 individual male flies from two reconstituted ancestral populations (replicate no. 27 and no. 28) and two evolved populations (replicate no. 4 and no. 9) were subjected to RNA-seq. The protocols of RNA extraction and library preparation are described in Lai and Schlötterer (2022). We access whole-body expression to interrogate all possible regulatory changes at the organismal level (including changes in allometry as well as cell type composition). We believe that this will be better translated to many organismal traits, but we acknowledge that tissue/single-cell expression profiles will inform us the acute transcriptional regulation.

### RNA-Seq Data Analysis for Mean and Variance Evolution

The processed RNA-seq data were obtained from Lai and Schlötterer (2022). To characterize the adaptive architecture of gene expression evolution, we compared the evolutionary dynamics of the variance between the genes with significant mean change and those without. The underlying assumption is that genes with significant mean expression changes are under selection and the rest of the transcriptome is not responding to the new environment (neutral). The genes with/without significant mean evolution for two evolved populations were taken from Lai and Schlötterer (2022).

We quantified the variance changes during adaptation for each gene by using the variance estimates of the expression (logCPM) of each gene in each population from (Lai and Schlötterer (2022). Briefly, raw read counts of each gene were normalized with the TMM method implemented in edgeR. We then applied natural log transformation to the expression of each gene (counts per million [CPM]) to fit normal assumption for all subsequent analyses and make mean and variance independent from each other.

Due to the moderate sample size, we performed additional checks for the uncertainty of variance estimates. Jackknifing was applied to measure the uncertainty of estimator (Fukunaga and Hummels 1989). The procedure was conducted independently on four populations, and we calculated the 95% confidence interval of the estimated variance (supplementary fig. S10, Supplementary Material online). Additionally, the robustness of variance estimation was also supported by the high correlation (rho = 0.79, Spearman's rank correlation) in variance estimates between the two independently reconstituted ancestral populations. The change of gene expression variance was determined by the *F* statistics calculated as the ratio between the variance within the ancestral population and the variance within the evolved population of each gene. To test whether selection on mean expression generally alters the expression variance, we compared the *F* statistics of genes with significant changes in mean expression to the genes without.

We note that the natural log transformation (as in Lai and Schlötterer (2022)) does not attempt to quantify and partition the noise due to the read sampling process at the gene level from the across-individual variance. Since we were primarily interested in the latter, we evaluated the effect of read sampling noise. We did an additional check by distinguishing the true biological variation and the measurement error (Eq. (1), from the user's guide of edgeR (Robinson et al. 2010)) of each gene using the statistical method implemented in edgeR, where the true variance across individuals (biological coefficient of variation [BCV]) of each gene can be estimated (tag-wised dispersion).


$$\mathrm{Total}\;CV^{2}=\mathrm{technical}\;CV^{2}+\mathrm{biological}\;CV^{2}\;(\mathrm{Eq}.\;(1))$$

The proportion of technical CV^2^ in total CV^2^ was calculated. We showed that the average proportion of technical CV^2^ gradually decreased with increasing sample size (supplementary fig. S3, Supplementary Material online), and for our sample size (*n* = 22), it is 17%. The correlation between BCV^2^ and the variance estimates after log transformation is 0.98 (Spearman's rho), suggesting that the two estimates are similar. As a sanity check, we repeated the comparisons of the variance changes in selected (DE) and neutral (non-DE) genes using BCV^2^. Similarly, the changes in variance of putative adaptive genes are indistinguishable from the genes that do not change their mean expression (*t*-test, *P*-value > 0.05; supplementary fig. S11, Supplementary Material online).

## Supplementary Material

evae077_Supplementary_Data

## Data Availability

All sequencing data are available in the European Nucleotide Archive (ENA) under the accession number PRJEB37011. Scripts for the analysis have been made available on the GitHub repository of this study (https://github.com/cloudweather34/simulation-evolution-of-phenotypic-variance).
