# Supplementary material for: Evolution of Phenotypic Variance Provides Insights into the Genetic Basis of Adaptation
Source: Genome Biol Evol. 2024 Apr 15;16(4):evae077. doi: 10.1093/gbe/evae077 (PMC11057206; doi:10.1093/gbe/evae077)
Supplement: evae077_Supplementary_Data [file evae077_supplementary_data.zip › supplementary_information_v21.docx]

**Supplementary text**

Genetic difference in gene expression across F1 families using single-fly sequencing

In this study, we included a prior experiment to evaluate how much of the expression variance can be explained by genetic variation. We aimed to use this analysis to demonstrate that single fly RNA sequencing can capture genetic difference rather than only noise.

Six out of 184 founder iso-female lines from the evolution experiment and were maintained for one generation with controlled egg density (400 eggs/bottle) in the same environment as the main experiment (12h 28°C with light followed by 12h 18 °C with dark conditions). Using the offspring, we generated three crosses between two of the six lines each: FL 138 x FL 137, FL 157 x FL 112, FL 123 x FL 127: we combined 50 virgin females from one of the lines with 50 males from the other line, let them lay eggs under density control as above and maintained and froze their F1 offspring in the same way as in the main CGE: sexes were separated after mating at the age of three days and snap-frozen at the age of five days at 2pm. From each cross, we used four F1 males to prepare individual RNA-Seq libraries as described above.

Natural log-transformation was applied to CPMs of all genes to improve data normality (Rocke and Durbin 2003). We found that three different families can be clearly distinguished by PCA (Supplementary figure SS1). This suggests that single fly RNA-seq can capture the genetic difference, rather than noise.

Simulating stabilizing selection without shift in trait optimum

It is possible that non-DE genes are subject to stabilizing selection without shift in trait optimum rather than neutrality. Hence, we performed additional computer simulations to evaluate the case under stabilizing selection without shift in trait optimum. We followed the parameter setting from the scenario with shape parameter 2.5 but the Gaussian fitness functions was changed into mean of $\bar{X_{anc.}}+0\sqrt{V_{anc.}}$ and standard deviation of $3.6\sqrt{V_{anc.}}$. We evaluate the power to distinguish the variance changes under stabilizing selection with and without shift in trait optimum with different numbers of contributing loci (M = 5, 25, 50, 100, 200 and 1000). We also investigate the correlation of variance changes between evolution replicates in the three different scenarios (stabilizing selection with shift in trait optimum, stabilizing selection without shift in trait optimum, and neutrality). When stabilizing selection operates, the change in variance should be correlated between replicate populations but such correlation is not expected under neutrality.

**
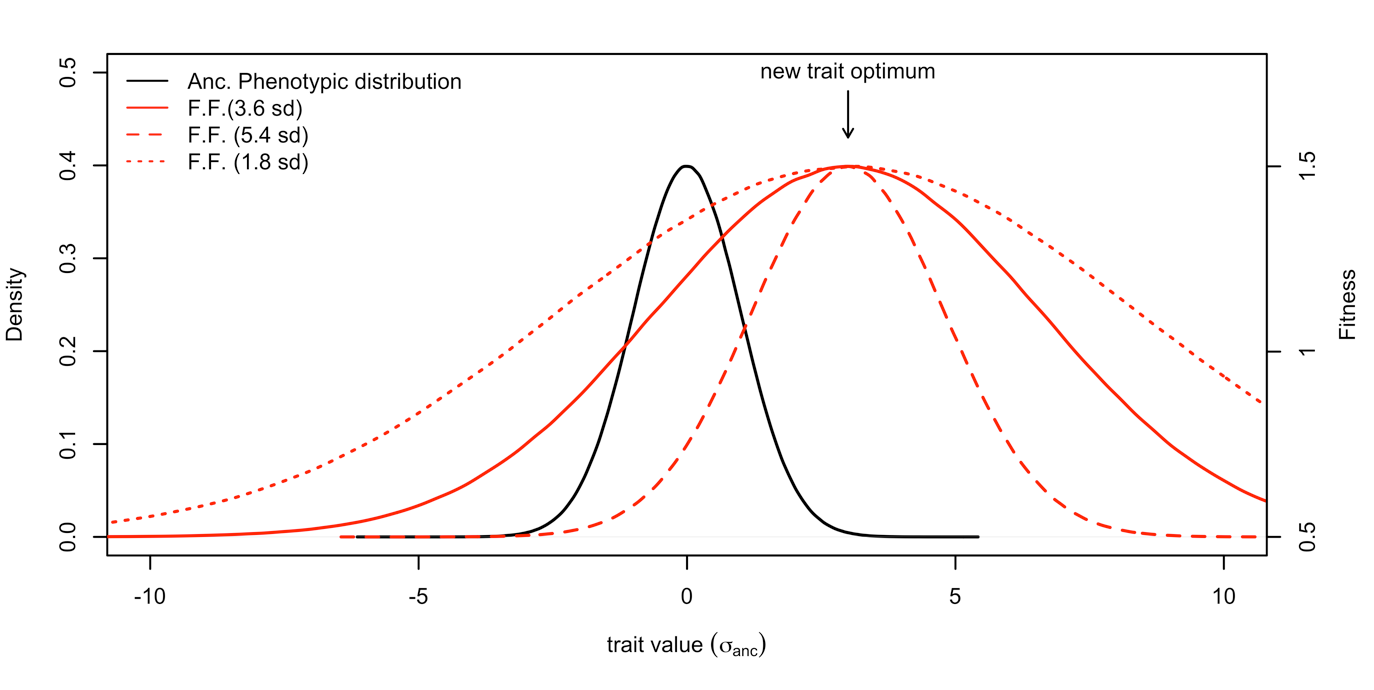
**

**Supplementary Figure S1. The evolutionary scenario for distant optimum shift.** We consider the case when a quantitative trait (in black) experiences a sudden shift in trait optimum under stabilizing selection. The imposed fitness functions (F.F.) are illustrated in red. The new trait optimum is set away from the ancestral trait mean by three standard deviations of the ancestral trait distribution for distal shift. To vary the strength of stabilizing selection, the variance of the fitness function is set as 1.8, 3.6 and 5.4 standard deviation of the ancestral trait distribution.


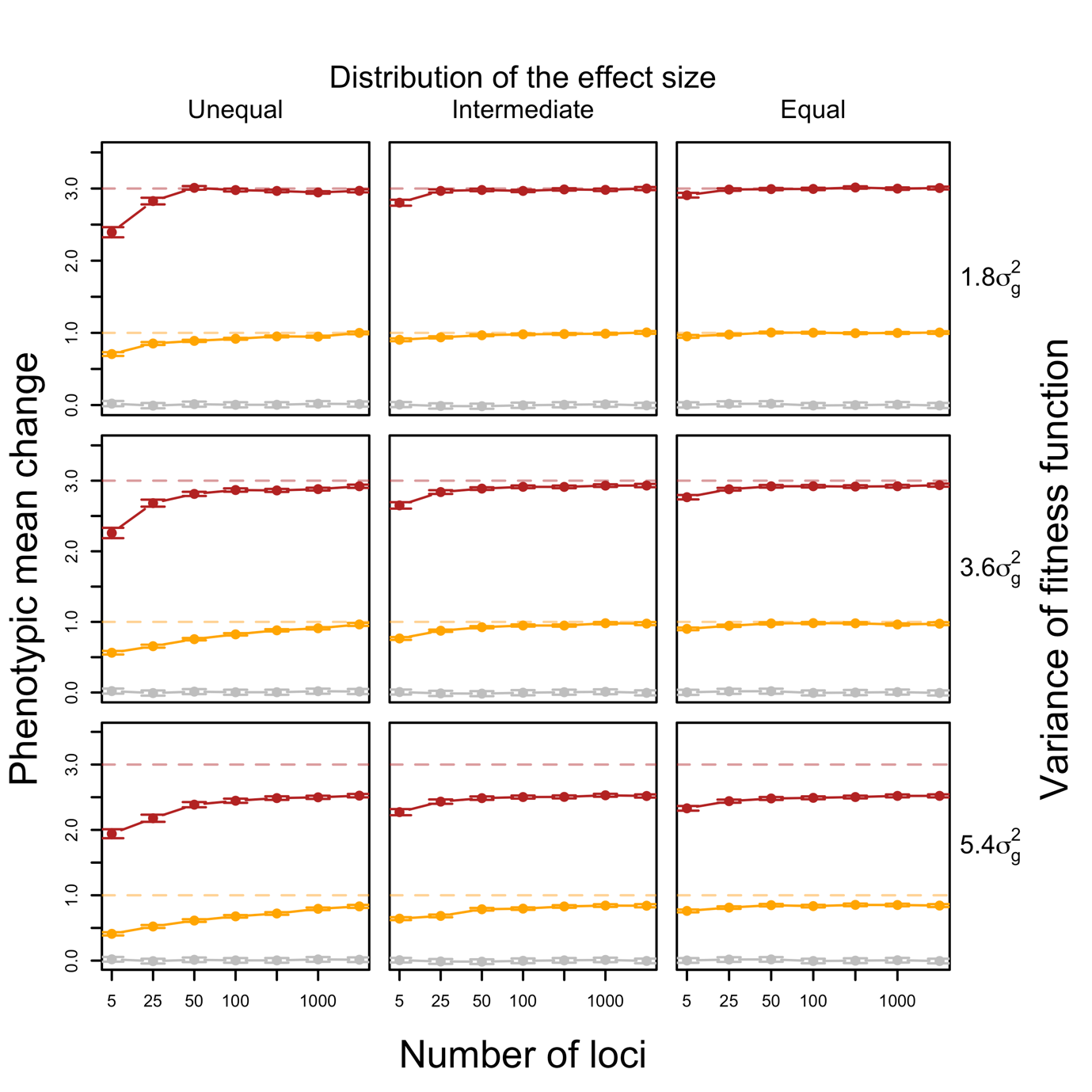


**Supplementary Figure S2. The changes in phenotypic mean when adapting to optimum shift.** The changes in phenotypic mean after 100 generations adapting to a mild/distant optimum shift (orange/red) are compared to the changes under neutrality (grey) on y axis. The changes in phenotypic mean are scaled by the standard deviation of the ancestral trait distribution. The simulations cover traits controlled by varying numbers of loci underlying the adaptation (x axes) with three different distributions of effect sizes (columns) and under different strength of stabilizing selection (rows). For each scenario, 1000 traits have been performed. In most cases, the traits under selection (orange/red) shift their means by one/three standard deviation of the ancestral trait distribution (i.e. reaching the new trait optimum) while the neutral traits (grey) stay unchanged. The error bar indicates the 95% confidence interval for 1000 simulated traits. The dash lines (orange/red) are the expected trait optimum at one/three standard deviation away.


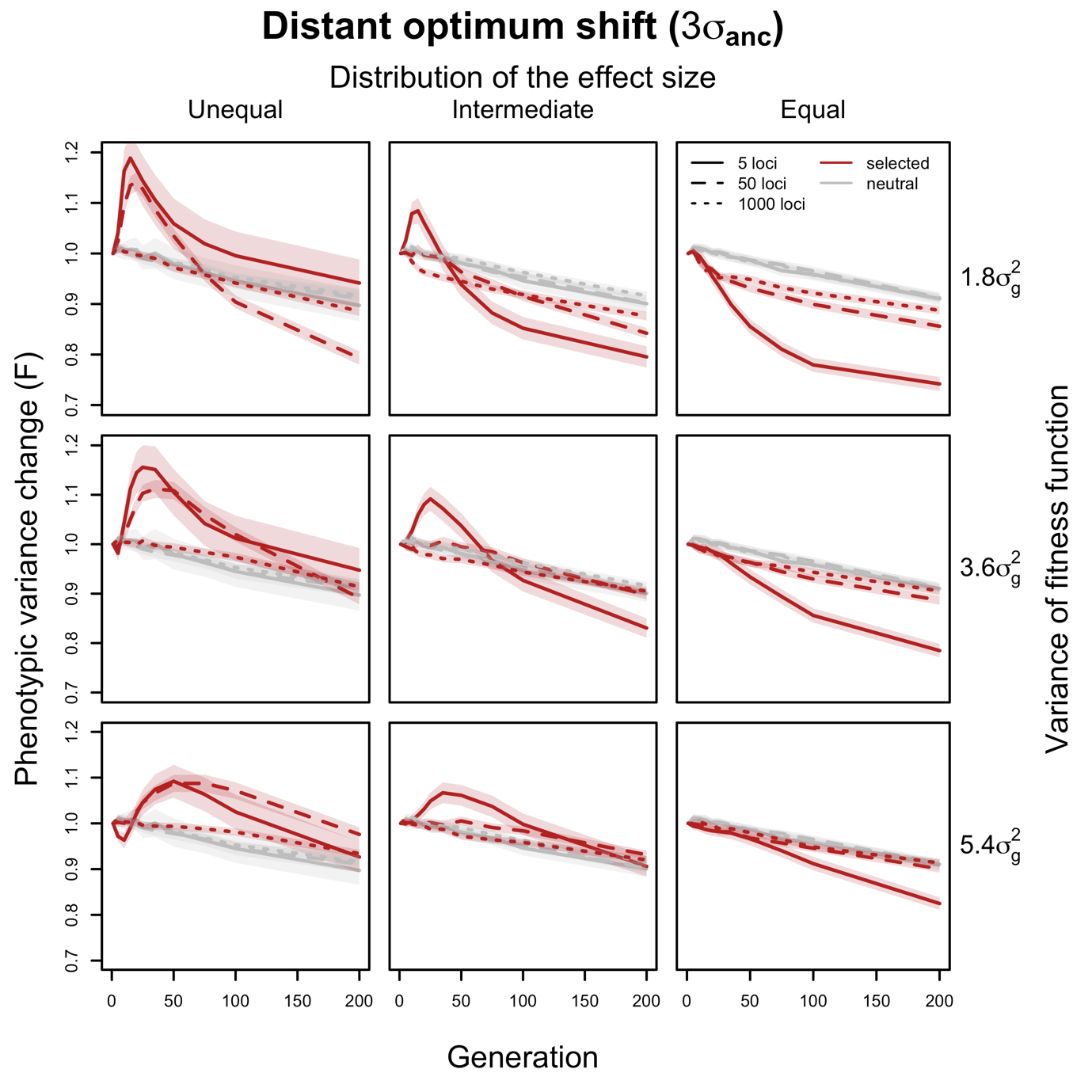


**Supplementary Figure S3. The trajectory of expected changes in phenotypic variance when adapting to a distant optimum shift.** The changes in phenotypic variance within 200 generations adapting to a distant optimum shift (red) are compared to the changes under neutrality (grey) on y axis. The average change in variance among 1000 traits (F) is calculated as the ratio of phenotypic variance between each evolved time point (generation x) and the ancestral state (${\sigma_{x}^{2}}/{\sigma_{1}^{2}}$). The translucent band indicates the 95% confidence interval for 1000 simulated traits. The simulations cover traits controlled by varying numbers of loci underlying the adaptation with three different distributions of effect sizes (columns) and under different strength of stabilizing selection (rows). For each scenario, we simulated 1000 traits. Only traits with the most (dotted lines, 1000 loci), intermediate (dash lines, 50 loci) and the least (solid lines, 5 loci) polygenic architectures are shown. Unlike the continuous decreasing pattern in the cases with mild optimum shifts, the variance of traits controlled by a few loci (5 loci) with largely dispersed effects would increase first and then decrease when the effect sizes of contributing loci are dispersed (red solid lines). Nevertheless, for traits with extremely polygenic basis, the phenotypic variance always stays stable over time (red dotted lines).


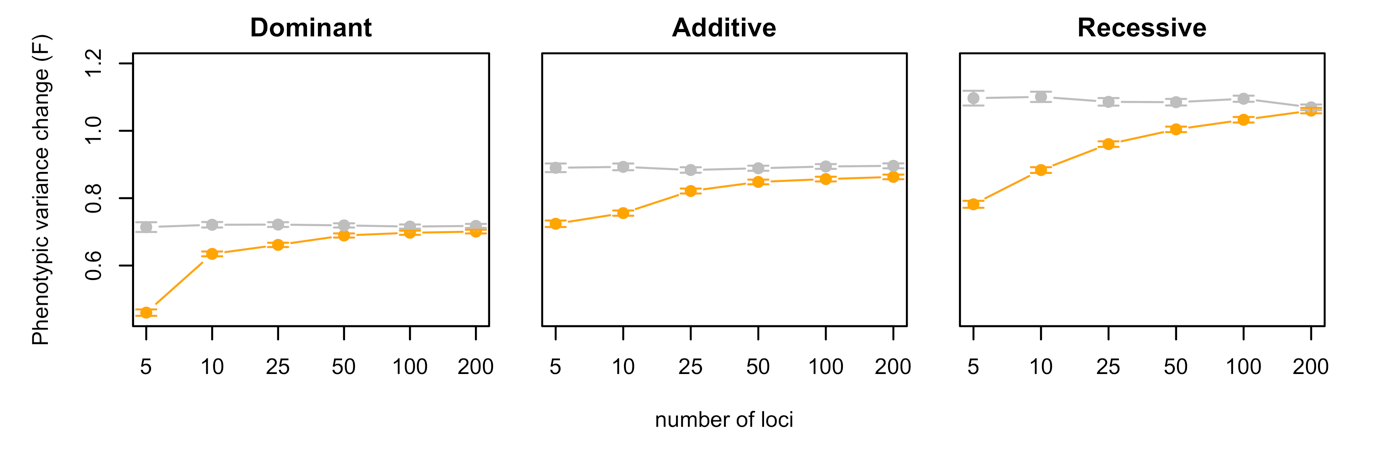


**Supplementary Figure S4. The expected changes in phenotypic variance for traits controlled by dominant and recessive alleles.** The changes in phenotypic variance after 100 generations adapting to a mild optimum shift (orange) are compared to the changes under neutrality (grey) on y axis. The change in variance (F) is calculated as the ratio between the evolved and ancestral phenotypic variance (${\sigma_{100}^{2}}/{\sigma_{0}^{2}}$). This simulation covers traits controlled by varying numbers of loci underlying the adaptation (x axes) with recessive, additive and dominant effects. For each scenario, we simulated 1000 traits. No matter how the dominance varies, the variance of the trait decreases drastically when the adaptation is controlled by a small number of loci. As the number of contributing loci increases, the phenotypic variance becomes more stable. The error bar indicates the 95% confidence interval for 1000 simulated traits.


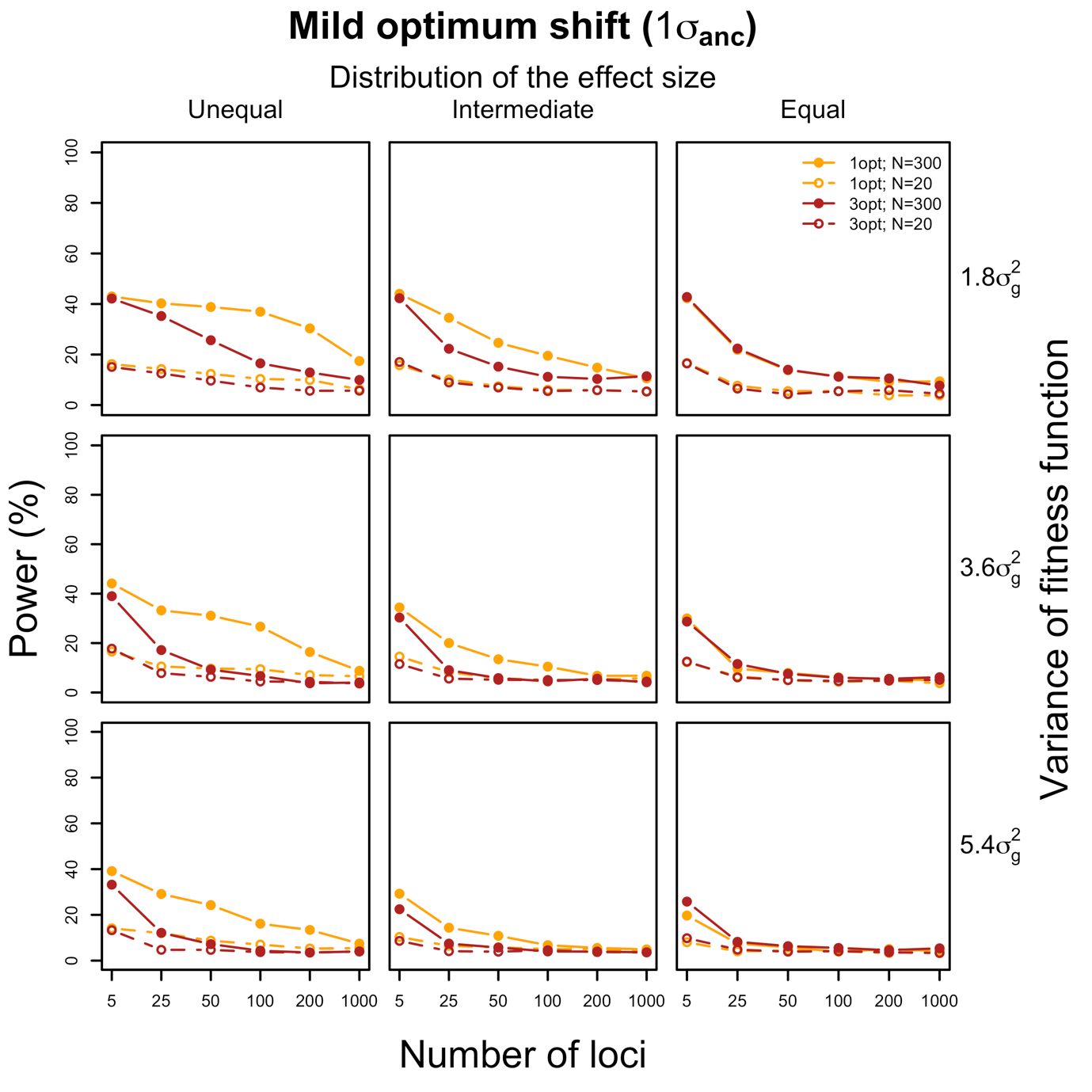


**Supplementary Figure S5. Power for detecting significant variance changes for a single trait.** With each genetic architecture (x-axis) under each selection scenario, we calculated how often we can detect a significant difference between the variance change of a single trait after 100 generations of selection and the neutral expectation (F of 0.9 according to the neutral simulations) (y-axis). The power is largely limited by the sample size. When all 300 individuals were considered, only 40% of power is observed under simple genetic control (5 loci). When the sampling processes of 20 individuals from the 300 simulated individuals was considered, there is nearly no power for the inference.

**
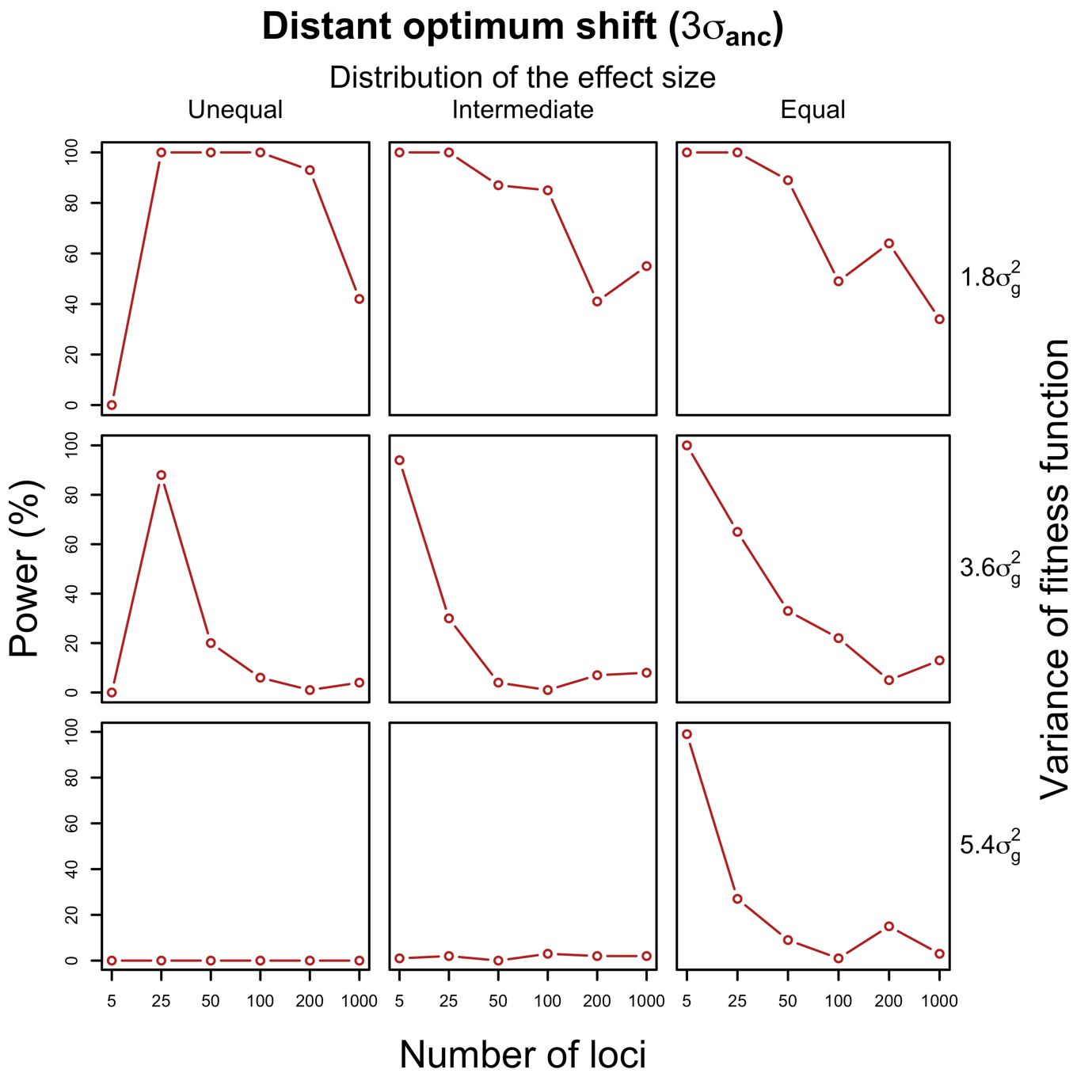
**

**Supplementary Figure S6. Power for detecting significant different variance change between a group of selected and neutral traits under distant optimum shift.** For each set of the 1000 traits controlled by different numbers of loci (x-axis) with varying effect sizes (columns) under each selection strength (rows), we calculated how often the variance changes of these 1000 selected traits differs from 1000 neutral traits after 100 generations (y-axis). The same genetic architecture is assumed for all 1000 selected traits with with a sample size of 20. In five out of the nine parameter combinations, we have nearly 100% power to detect a significant difference when all traits were under simple genetic control (5 loci). The power gradually decreases when the number of loci increased. Nevertheless, in the other parameter combinations, exception can be found for the traits controlled by a few loci (5 loci) with largely dispersed effects (see Discussion).


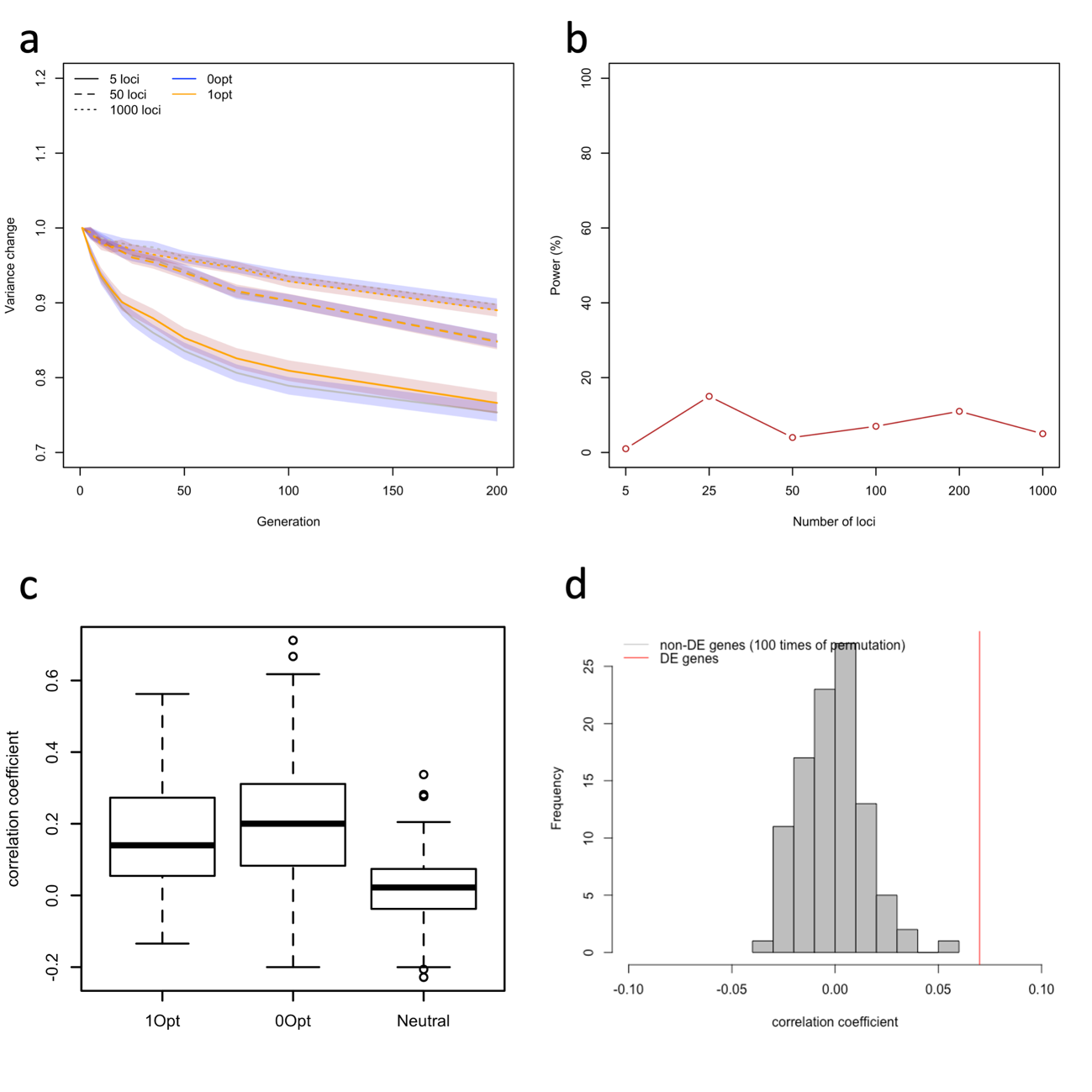


**Supplementary Figure S7. Stabilizing selection without shift in trait optimum. a.** Changes in phenotypic variance (y-axis) across 200 generations (x-axis) of evolution with stabilizing selection either with a shift in trait optimum ($1\sigma_{anc}^{2}$; orange) or without ($0\sigma_{anc}^{2}$; blue). The average change in variance of 1000 traits (F) is calculated as the ratio of the phenotypic variance between each evolved time point (generation x) and the ancestral state. The translucent band indicates the 95% confidence interval for 1000 traits. The simulated traits are controlled by a different number of loci (5 loci, 50 loci and 1000 loci). **b.** Power to detect a significantly different variance change between a group of traits with ($1\sigma_{anc}^{2}$) and without ($0\sigma_{anc}^{2}$) shift in trait optimum for a different number of loci (x-axis). **c.** Correlation of variance changes in two populations evolved under stabilizing selection with shift in trait optimum ($1\sigma_{anc}^{2}$), without shift in trait optimum ($0\sigma_{anc}^{2}$) and under neutrality. The correlation under stabilizing selection is very similar independent of whether a shift in trait optimum is assumed (p-value > 0.05), but much higher than the correlation under neutrality (p-value < 0.05). **d.** Observed correlation of variance change between two evolved replicates for DE and non-DE genes in the empirical data. Pearson’s correlation coefficient of variance changes (log(F)) for DE genes across two evolution replicates was calculated (r=0.07; red line). The correlation coefficient is significantly higher than the variance changes for the non-DE genes when we down sampled the non-DE genes to the number of DE genes (n=4,323) (grey bar; 100 permutations) (p-value < 0.01).


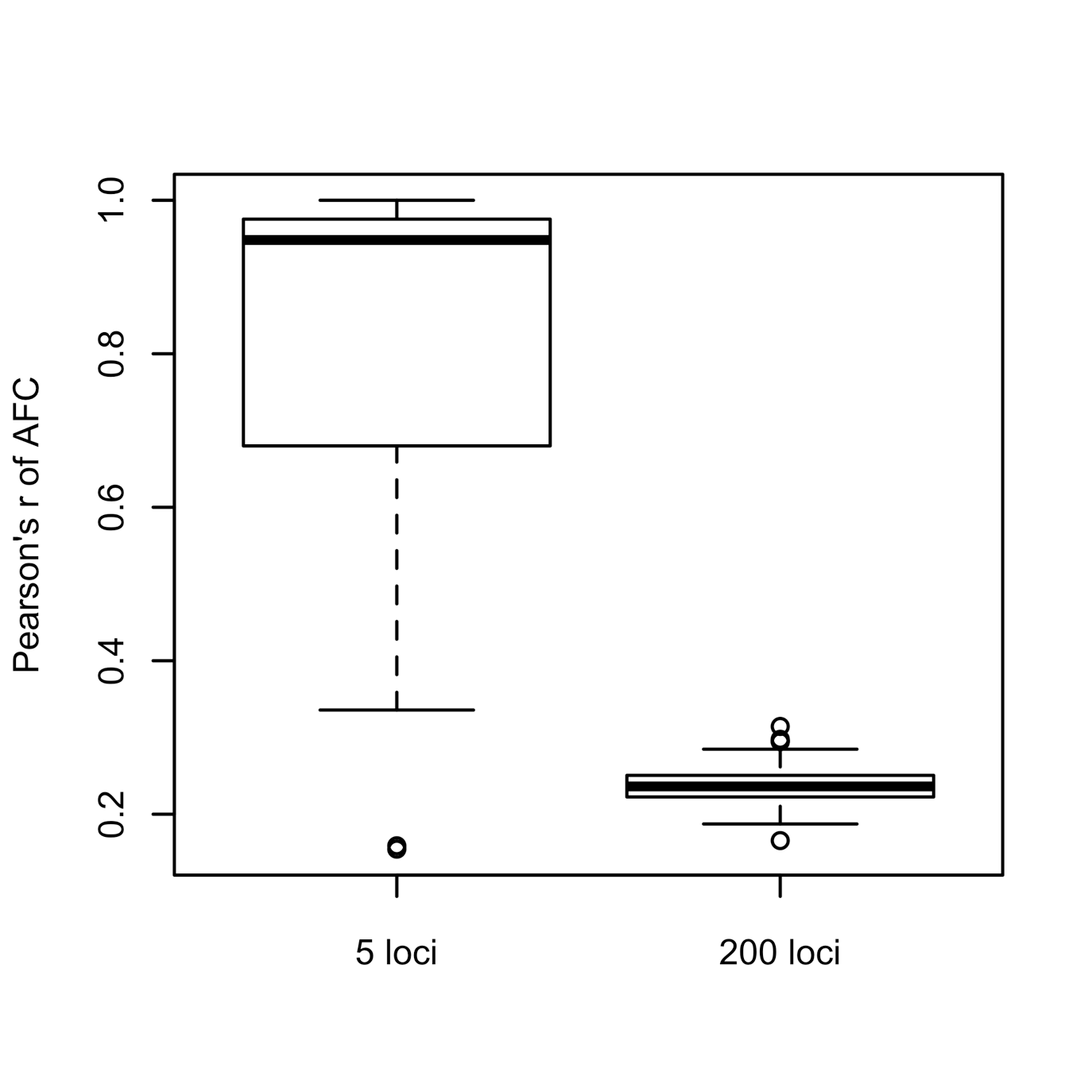
 **Supplementary Figure S8. Parallelism in genomic response across 10 evolved replicates for traits under the control of five loci and 100 loci.** For the loci with allele frequency change of at least 10% in 100 generations, the average Pearson’s correlation coefficient of the frequency change between all pairs of two evolved replicates was calculated to describe the parallelism of the evolution at these loci. An average across loci is used to obtain a general parallelism. 100 traits with 10 evolution replicates have been performed for each scenario. With five contributing loci, the genomic evolution of the contributing loci is more parallel across the 10 evolved replicates compared to the case when with 100 contributing loci.


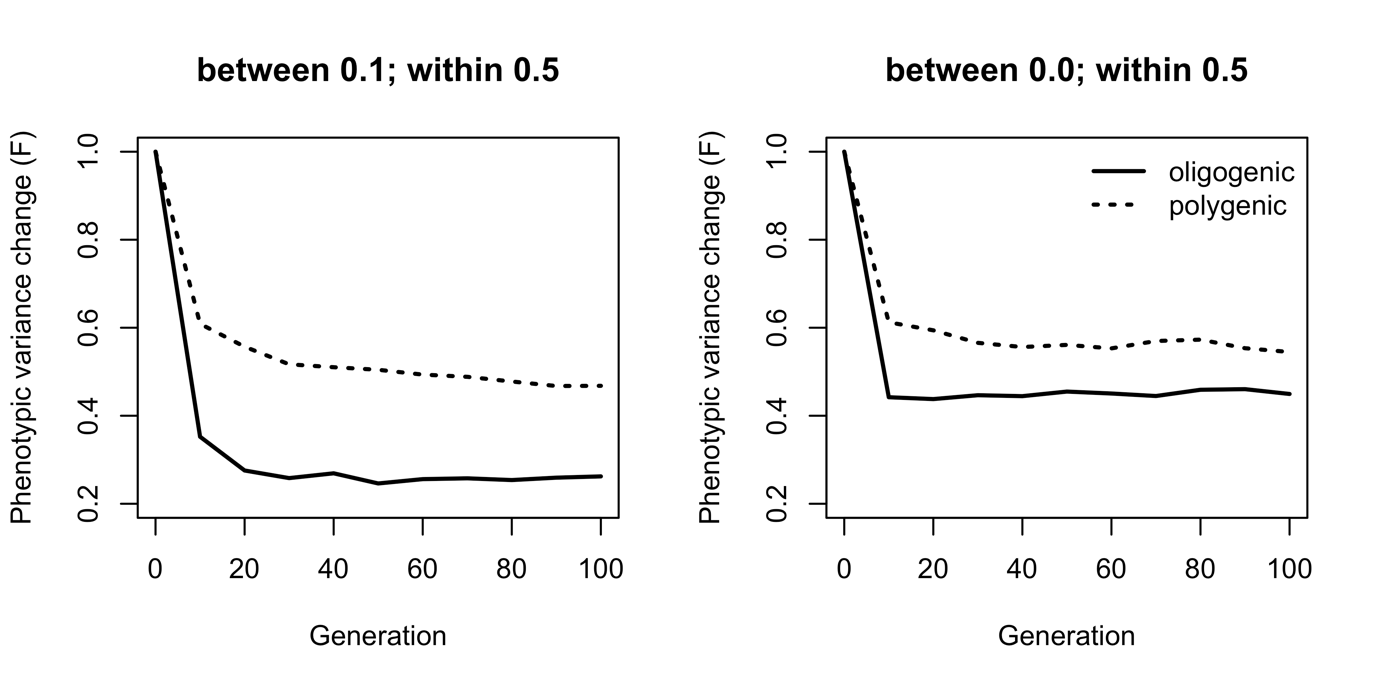


**Supplementary Figure S9. The variance dynamics of selected traits regulated by connected modules.** We monitored the average variance changes relative to ancestral of 1,000 simulated traits under fully connected modular regulation (50 modules of 20 expression traits) within 100 generations of adaptation. The number of contributing loci to the modules were varied to simulate oligogenic (solid lines; < 60 loci) and polygenic (dash lines; < 300 loci) architectures. The effect size of each contributing loci was drawn from a multivariate normal distribution. The contributing loci affect all simulated expression traits while traits within a module exhibit stronger genetic correlation (covariance of 0.5) than the ones in different modules (covariance of 0 or 0.1). Considering fully connected modularity, even polygenic traits drop in variance overtime while the difference between oligogenic and polygenic architectures holds.

**
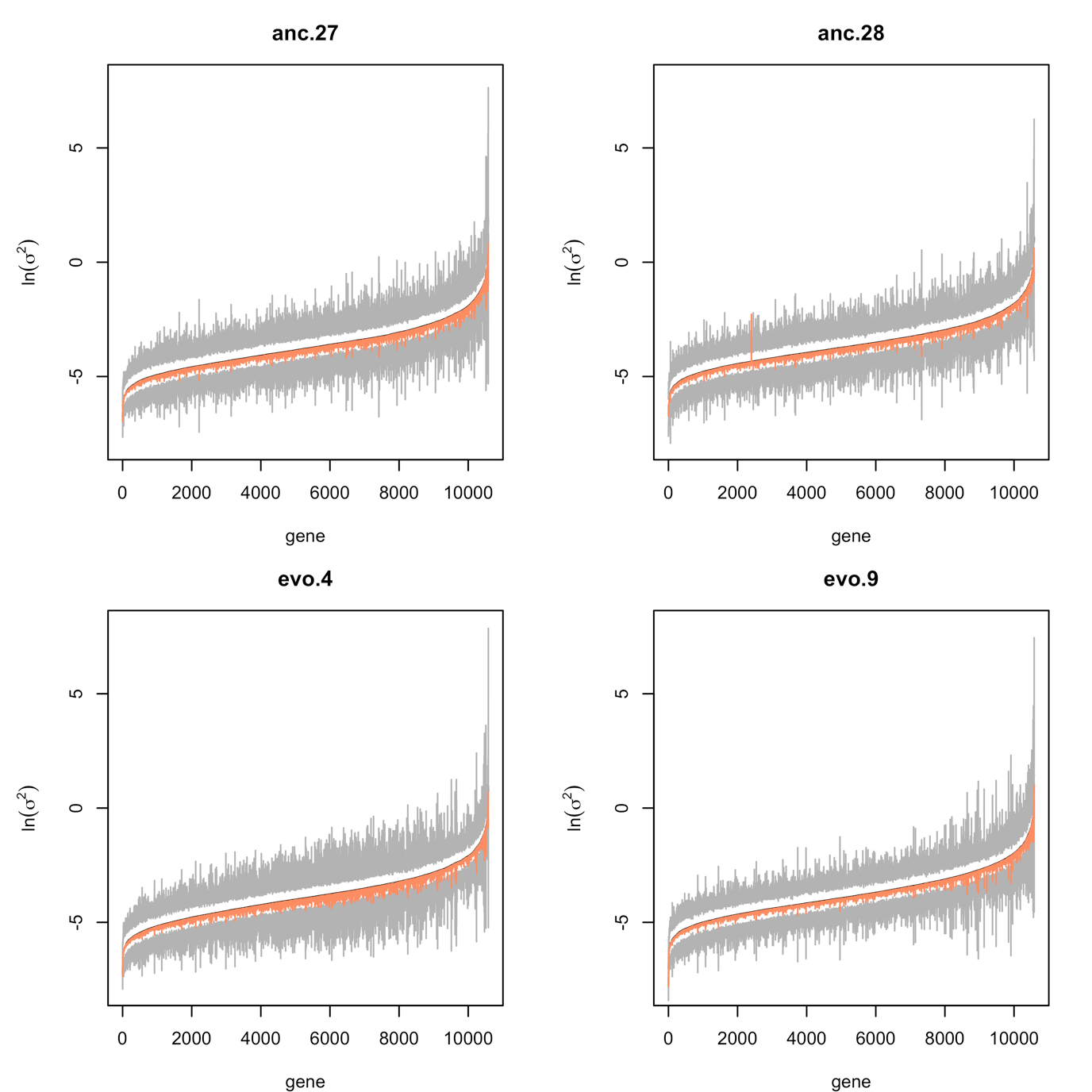
**

**Supplementary Figure S10. Robustness of the variance estimation using individual sequencing data.** Jackknife method was applied to measure the uncertainty of variance estimation within each population. Given a sample size of K, the procedure is to estimate the variance of each gene for K times, each time leaving one sample out. The procedure was conducted independently on 4 populations (anc.27, anc.28, evo.4 and evo.9). In each panel, we visualize Jackknife approximated 95% confidence interval for the variance estimates of each gene. The genes are ordered based on the average variance estimates (black dash line) on the x-axis. The upper and lower limits of the 95% confidence interval are indicated with grey curves. The salmon line denotes the observed value of the variance estimates. In most cases, the estimates lie in the confidence interval, suggesting robust estimation.

**
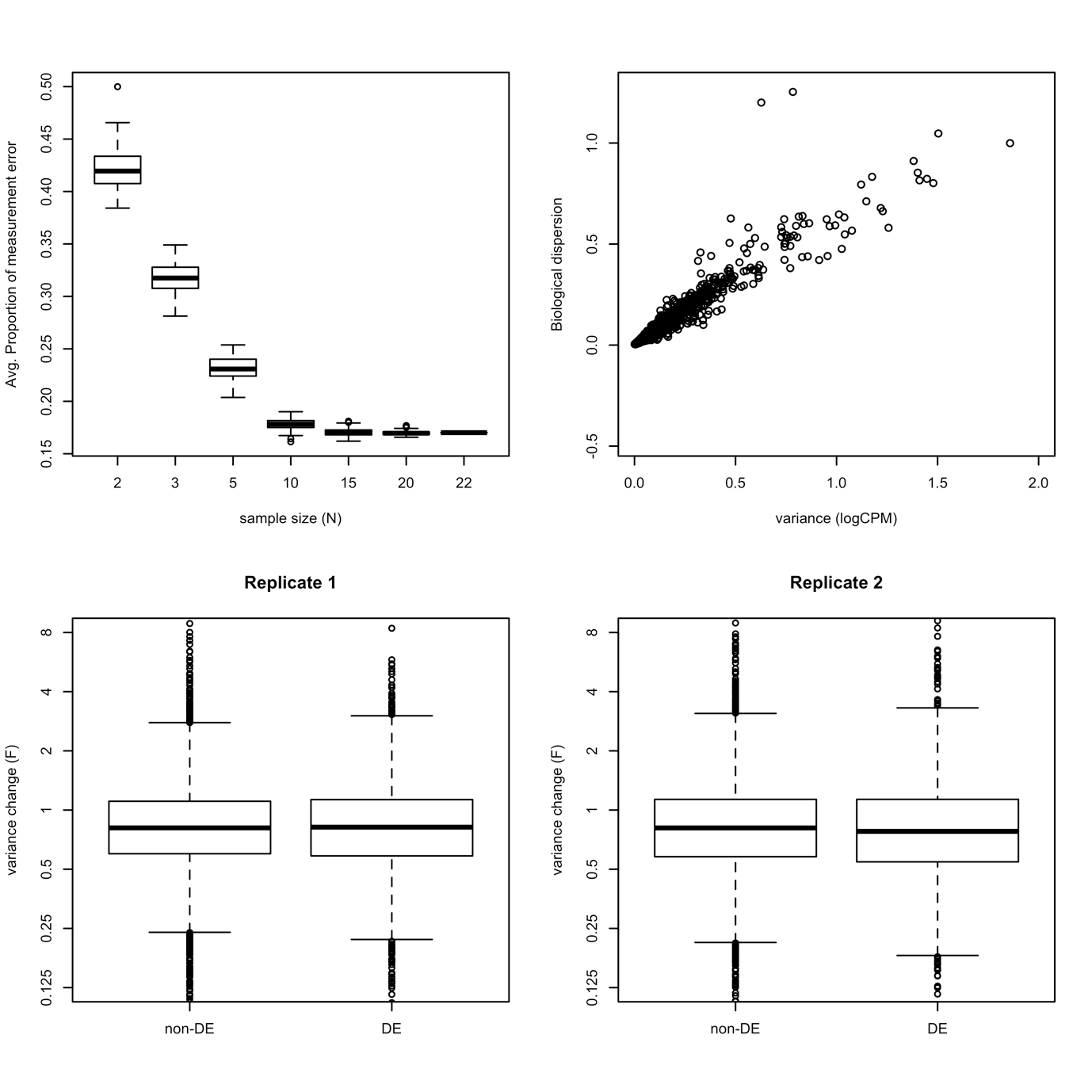
**

**Supplementary Figure S11. The decomposition of measurement error in RNA-Seq data and its implication for the tests of variance evolution. (A)** The relationship between the average proportion of measurement error among the RNA-Seq samples and the sample sizes is visualized. As the sample size increases, the measurement error of RNA-Seq data is well controlled. The results are consistent across replicate populations. For simplicity, only the result in the first replicate of ancestral population is shown. **(B)** We observed a strong correlation between the variance estimates after log-transformation and the biological coefficient of variation (BCV) estimated from edgeR, which claims to exclude the measurement error. Such strong correlation suggests that measurement error in RNA-Seq data wouldn’t have strong impact on the test of variance of evolution. The results are consistent across replicate populations. For simplicity, only the result in the first replicate of ancestral population is shown. **(C)** Using the BCV estimates, we repeated the comparison between the variance changes ($F=\frac{{BCV}_{evo}^{2}}{{BCV}_{anc}^{2}}$) of the genes with and without significant evolution in mean expression. In both replicates, the distribution of variance changes is indistinguishable between DE genes and non-DE genes (t-test, p > 0.05 for both replicates).


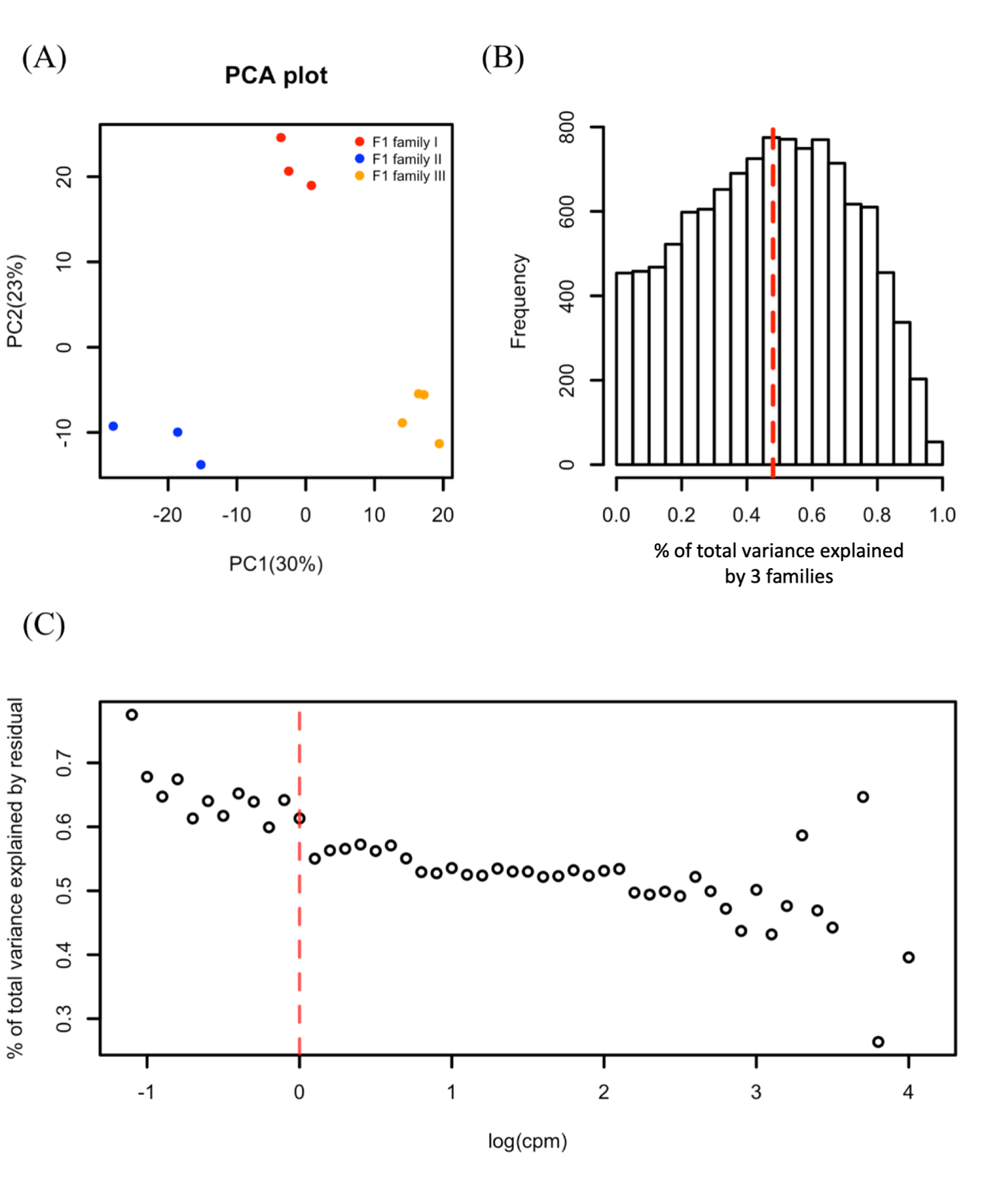


**Supplementary Figure SS1. Genetic variance in gene expression across F1 families.** Principal component analysis (PCA) on the transcriptomes of F1 individuals from three different crosses between the founder iso-female lines. Individuals from different families clustered nicely based on the first two PCs.

**Titles and legend for supplementary files**

**Supplementary file 1. Library information of the sample in this study.** This file provides a list of all sequenced samples and the library information.

**References**

Ayroles J. F., M. A. Carbone, E. A. Stone, K. W. Jordan, R. F. Lyman, *et al.*, 2009 Systems genetics of complex traits in Drosophila melanogaster. Nat. Genet. 41: 299–307. https://doi.org/10.1038/ng.332

Rocke D. M., and B. Durbin, 2003 Approximate variance-stabilizing transformations for gene-expression microarray data. Bioinformatics 19: 966–72.
